# Genome-Wide DNA Methylation Patterns of Bovine Blastocysts Developed *In Vivo* from Embryos Completed Different Stages of Development *In Vitro*

**DOI:** 10.1371/journal.pone.0140467

**Published:** 2015-11-04

**Authors:** Dessie Salilew-Wondim, Eric Fournier, Michael Hoelker, Mohammed Saeed-Zidane, Ernst Tholen, Christian Looft, Christiane Neuhoff, Urban Besenfelder, Vita Havlicek, Franca Rings, Dominic Gagné, Marc-André Sirard, Claude Robert, Habib A. Shojaei Saadi, Ahmed Gad, Karl Schellander, Dawit Tesfaye

**Affiliations:** 1 Institute of Animal Science, Animal Breeding and Husbandry Group, University of Bonn, 53115 Bonn, Germany; 2 Centre de recherche en biologie de la reproduction, Faculté des sciences de l’agriculture et de l’alimentation, INAF, Pavillon des services, Université Laval (Québec), Canada G1V 0A6; 3 Institute of Animal Breeding and Genetics, University of Veterinary Medicine Vienna, A-1210, Vienna, Austria; 4 Department of Animal Production, Faculty of Agriculture, Cairo University, 12613, Giza, Egypt; University of Connecticut, UNITED STATES

## Abstract

Early embryonic loss and altered gene expression in in vitro produced blastocysts are believed to be partly caused by aberrant DNA methylation. However, specific embryonic stage which is sensitive to in vitro culture conditions to alter the DNA methylation profile of the resulting blastocysts remained unclear. Therefore, the aim of this study was to investigate the stage specific effect of in vitro culture environment on the DNA methylation response of the resulting blastocysts. For this, embryos cultured in vitro until zygote (ZY), 4-cell (4C) or 16-cell (16C) were transferred to recipients and the blastocysts were recovery at day 7 of the estrous cycle. Another embryo group was cultured in vitro until blastocyst stage (IVP). Genome-wide DNA methylation profiles of ZY, 4C, 16C and IVP blastocyst groups were then determined with reference to blastocysts developed completely under in vivo condition (VO) using EmbryoGENE DNA Methylation Array. To assess the contribution of methylation changes on gene expression patterns, the DNA methylation data was superimposed to the transcriptome profile data. The degree of DNA methylation dysregulation in the promoter and/or gene body regions of the resulting blastocysts was correlated with successive stages of development the embryos advanced under in vitro culture before transfer to the in vivo condition. Genomic enrichment analysis revealed that in 4C and 16C blastocyst groups, hypermethylated loci were outpacing the hypomethylated ones in intronic, exonic, promoter and proximal promoter regions, whereas the reverse was observed in ZY blastocyst group. However, in the IVP group, as much hypermethylated as hypomethylated probes were detected in gene body and promoter regions. In addition, gene ontology analysis indicated that differentially methylated regions were found to affected several biological functions including ATP binding in the ZY group, programmed cell death in the 4C, glycolysis in 16C and genetic imprinting and chromosome segregation in IVP blastocyst groups. Furthermore, 1.6, 3.4, 3.9 and 9.4% of the differentially methylated regions that were overlapped to the transcriptome profile data were negatively correlated with the gene expression patterns in ZY, 4C, 16C and IVP blastocyst groups, respectively. Therefore, this finding indicated that suboptimal culture condition during preimplantation embryo development induced changes in the DNA methylation landscape of the resulting blastocysts in a stage dependent manner and the altered DNA methylation pattern was only partly explained the observed aberrant gene expression patterns of the blastocysts.

## Introduction

In vitro embryo production (IVP) using oocytes matured and fertilized under in vitro culture condition has been a common practice for commercial and research purposes. Despite years of optimization, early embryonic losses, placental dysfunction, fetal death and over sized fetuses are still observed in embryos produced under in vitro culture conditions [1,2]. In addition, in vitro originated embryos are marked by alterations in their transcriptome profile compared to their in vivo counterparts [3–8] and this altered gene expression could be partly due to epigenetic reprogramming errors caused by aberrant DNA methylation [2].

DNA methylation is believed to be one of the mechanisms involved in regulating the gene expression profile of the embryo during its subsequent development. DNA modifications resulted from the addition of a methyl (CH_3_) group to the cytosine residue in CpG dinucleotides within the DNA sequence by the catalytic activity of *DNMT* enzymes [9,10] make the DNA to be less accessible to the transcription machinery, consequently hindering the gene expression [11].

Unlike differentiated somatic cells whose genomic methylation patterns seem to be stable, the DNA methylation pattern in germ cells and preimplantation embryos is changing dynamically to maintain cell reprograming during development [10]. For instance, prior to fertilization, the sperm genome is relatively methylated while the oocyte genome is hypomethylated [10,12], but the methylation level in the CpG islands may be greater in oocyte compared to sperm. In mouse, the paternal genome undergoes active genome-wide demethylation immediately after fertilization, while demethylation of the maternal genome happens in a sequential fashion [13]. On the other hand, another study indicated the presence of active methylation in the paternal and maternal genomes during embryonic development [14], while others reported stable methylation levels in the early cleavage stages of embryos [12].

In bovine, the DNA methylation pattern changes dynamically in a sex and stage dependent manner during preimplantation embryo development [15]. For instance, while a dramatic loss of DNA methylation occurs in the male pronucleus immediately after the union of gametes [16], higher DNA methylation appears at the 8-cell stage in the female embryos and at the blastocyst stage in male embryos [15].

Apart from sex and stage of development, the DNA methylation pattern of the embryo is also modified by the culture environment [17]. Previous reports indicated altered placental DNA methylation patterns in human pregnancy established after transfer of in vitro produced embryos [18]. Similarly, a study in rabbit embryos has also shown faster demethylation in in vitro embryos compared to their in vivo counterparts [19]. Altered DNA methylation patterns in the fetal liver due to changes in oocyte maturation media and embryo culture have also been reported [20]. Moreover, in vitro conditions during preimplantation period could cause epigenetic abnormalities in the placenta during the later stages of pregnancy [21]. All in all, previous data indicated that suboptimal culture conditions may induce aberrant DNA methylation patterns during the preimplantation stage and fetal development. However, the preimplantation embryonic stages which are sensitive to culture conditions to cause significant modifications of the DNA methylation patterns in the resulting blastocysts remained unclear. In our previous study, blastocysts derived from in vitro embryos transferred to the recipient animals before or after embryonic genome activation displayed altered transcriptome profiles compared to the blastocysts developed completely in the vivo conditions [22]. Therefore, we hypothesized that stage specific suboptimal culture condition during preimplantation embryo development could cause significant modifications of the DNA methylation in the resulting blastocysts. Therefore, the main objective of this study was designed to explore the global DNA methylation patterns of the blastocysts derived from embryos cultured under in vitro culture conditions in a stage specific manner before being transferred to the in vivo condition.

## Materials and Methods

### Animal handling, management and estrous synchronization

Simmental healthy heifers were selected for this experiment. All experimental heifers received similar total mixed ration, and they were kept in the same farm and housing conditions. Handling and management of experimental animals were adhered to the rules and regulations of the German law of animal protection. Moreover, the experiment was approved by the Animal Welfare committee of the University of Bonn with proposition number 84–02.05.20.12.075.

### *In vitro* embryo production, embryo transfer and flushing

Oocytes were collected from bovine ovaries obtained from local abattoirs. For this, cumulus-oocyte complexes (COCs) were aspirated from follicle size of 2 to 8 mm diameter. Good quality oocytes were matured and fertilized in vitro as described previously [7,8,23]. After 18 hrs of oocytes and sperm co-incubation, the cumulus cells were removed and cumulus-free presumptive zygotes were washed three times in CR1aa supplemented with 10% heat inactivated estrous cow serum. The embryos were then cultured in 400 μl of culture medium in four well dishes (Nunc, Roskilde, Denmark) covered with mineral oil at 38.7°C in 5% CO_2_ in humidified air. Some of the zygotes were then transferred to the recipient animals and others were transferred at 4-cell or 16-cell and other group of zygotes in vitro cultured until blastocysts stage. Therefore, for this study five blastocyst groups were generated. The first (ZY), second (4C) and the third (16C) blastocyst groups were obtained from embryos developed in vivo after transfer of in vitro developed zygotes, 4-cell and 16-cell stage embryos, respectively to estrous synchronized recipients using transvaginal endoscopic tubal transfer technique and the blastocysts were collected at day 7 of the estrous cycle by uterine flushing. Estrous synchronization was performed as indicated previously in our lab [22,24]. The fourth (IVP) blastocyst group was obtained from zygotes culture under in vitro condition until the blastocysts stage. The number of zygotes, 4-cell or 16-cell stage embryos transferred and the blastocysts recovered from each embryo group is indicated in S1 Table. The fifth (VO) blastocyst group was obtained from embryos developed completely under in vivo conditions. For this, Simmental heifers were estrous synchronized, super ovulated and artificially inseminated using the semen collected from the bull whose semen was used for in vitro fertilization as described previously [8,22,24] and the blastocyst stage embryos were collected 7 days post insemination as described above by uterine flushing. A total of four pools of blastocysts each consisting of 10 blastocysts from ZY, 4C, 16C, IVP and VO groups were used for global DNA methylation analysis and additional three pools each consisting of 10 blastocysts were generated to validate the array result using bisulfite sequencing.

### Genomic DNA isolation and DNA fragmentation

Genomic DNA was isolated from all blastocyst groups using the Allprep DNA/RNA micro kit (Qiagen) according to the manufacturer’s protocol. The genome DNA was then fragmented using the MseI enzyme in the presence of 0.5 μl bovine serum albumin (100x), 10 μl NEB buffer 4 (10x) and spike-in controls at 37°C for 16 hrs followed by further incubation at 65°C for 20 min. After the end of incubation period, gDNA was precipitated using linear acrylamide (Ambion), sodium acetate (3M, pH 5.2) and ethanol. After repeated washing in 70% ethanol, the gDNA pellets were dissolved in 5 μl nuclease free water.

### Adapter ligation and methyl sensitive enzymes digestion

The protocols regarding adapter/linker ligation and methyl sensitive enzyme cleavage have been previously described in Shojaei Saadi et.al [25]. Briefly, MseLig12 (100 μM) (5'-TAA CTA GCA TGC-3') and 0.5 μl MseLig21 (100 μM) (5-AGT GGG ATT CCG CAT GCT AGT-3') adapters were ligated at the fragmented gDNA sites in the presence of 0.5 μl 10× One-Phor-All Buffer PLUS (Pharmacia Biotech) and 1.5 μl nuclease free water. The reaction was incubated at 65°C for 20 min to initiate annealing and the temperature was decreased to 15°C at the rate of 1°C /min. Following this, 1 μl ATP (10 mM) and 1 μl T4 DNA Ligase (5U/μl) was added to the reaction and incubated for 16 hrs at 15°C. At the end of reaction, unmethylated genomic sites within the MseI-MseI region of the fragmented were cleaved by DNA FastDigest™ methyl-sensitive restriction endonucleases enzymes (HpaII, HinP1I and AciI). For this, a mix consisting of 5μl of FastDigest buffer (10X), 0.5 μl HpaII (10U/μl), 0.5 μl HinP1I and 34 μl nuclease free water was added to the reaction and incubated overnight at 37°C. Afterwards, a mix consisting of 0.5 μl AciI, 5.0 μl of FastDigest buffer 10X and 44.5 μl of water was added to the reaction and further incubated at 37°C for 4 hrs. The reaction was then terminated after inactivating the enzyme at 80°C for 10 min.

### Confirmation of methyl sensitive cleavage

DNA cleavage by methyl sensitive enzyme was confirmed by analyzing the spike-in controls added during gDNA fragmentation step as previously described in the EmbryoGENE DNA Methylation Array (EDMA) Platform [25]. For this, qPCR was preformed by preparing separate mixes consisting of 0.5 μl forward and 0.5 μl reverse primers (10 μM) (targeting HpaII, HinP1I or AciI sites), 1.6 μl MgCl_2_ (25 mM), 2μl LC master Fast start mix (10x) and 14.4 μl Nuclease free water. Undigested spike-in (1/1000 dilution) and non-template controls were used as positive and negative controls, respectively. The amplification plot and the dissociation curve were evaluated for each of the HpaII, AciI or HinP1I primer sets. The differences in threshold cycle (Ct) between protected (hypermethylated) and unprotected (unmethylated) control templates were determined and uniformity of the samples was evaluated based on the qPCR amplification curves. Samples with the threshold cycle (Ct) ≥ 5 compared to undigested controls and displaying uniformity with other samples cleaved > 97% in the same cohort were used for downstream treatment. Insufficiently cleaved samples were subjected to a second treatment with specific methyl sensitive restriction enzyme.

### Genomic DNA amplification

After methyl sensitive enzyme cleavage, the gDNA was subjected to ethanol precipitation and the resulting gDNA pellets were dissolved in 10 μl nuclease free water. The gDNA was then subjected to two rounds of ligation-mediated polymerase chain reaction (LM-PCR) as indicated previously [25,26]. After PCR amplification, 5 μl of the PCR product was loaded onto 3% ethidium bromide stained agarose gel electrophoresis to confirm the distribution of the digested gDNA. Once the presence of several bands were observed, further amplification was performed using 0.75 μl of amplified DNA to which a mix consisting of 0.75 μl 100 μM MseLig21, 5μl PCR buffer 1 Roche (17.5mM MgCl2), 4μl dNTPs, 0.8 μl Roche DNA long template enzyme mix and 38.7μl water was added. Amplification reaction was performed in four steps, in first step samples were incubated at 94°C for 60 min, 65°C for 30 s and 72°C for 2 min followed by 14 cycles at 94°C for 40 s, 65°C for 30 s and 72°C for 90 s. The reaction continued for 9 cycles at 94°C for 40 s, 65°C for 30 s and 72°C for 2 min and finally terminated after incubating at 72°C for 5 min. The PCR products were then purified using PCR purification kit (Qiagen) and finally eluted in 30 μl of 1/10 buffer elution buffer. Following this, the gDNA was incubated in the presence of 10 μl of buffer 4 NEB (10X), 1 μl BSA (100x), 1 μl Msel (10 u/μl) and 48 μl water at 37°C followed by incubation at 65°C for 20 min. At the end of reaction, the samples were purified using PCR purification kit (Qiagen) and finally eluted in 30 μl of 1/10 elution buffer. The quantity and quality of amplified digested gDNAs were measured using NanoDrop 1000 spectrophotometer.

### Dye labeling and hybridization

Two micrograms amplified gDNA from each blastocyst sample was labeled with either Cy-3 or Cy-5 dyes using Universal Linkage System Fluorescent gDNA labelling kit (Kreatech Biotechnology) at 85°C for 30 min. The samples were then purified using the Qiagen PCR purification kit (Qiagen). The dye incorporation and DNA concentration were measured using NanoDrop 1000 spectrophotometer. Hybridizations were performed by mixing 1 μg of labeled gDNA sample of ZY, 4C, 16C or IVP, 1 μg of labeled gDNA of the VO blastocyst group, 25 μl of bovine Cot-1 DNA (1.0 mg/mL, Applied Genetics Laboratories), 2.6 μl of Agilent 100x Blocking Agent and 130 μl of Agilent 2x HI-RPM Hybridization Buffer (Agilent Technologies). The samples were incubated at 95°C for 3 min, at 37°C for 30 min and then 65 μl of Agilent-CGHBlock was added to each of the samples. The samples were loaded onto the microarray and hybridization was preformed in a hybridization oven (Shel Lab) for 40 hrs at 65°C and 20 rpm. Four biological replicates of ZY, 4C, 16C or IVP blastocyst group were hybridized to the four biological replicates of VO group in EmbryoGENE DNA Methylation Array EDMA which harbors nearly 2.3 M CpG sites corresponding to 10% of the bovine CpG sites in the bovine genome [25]. At the end of hybridization, the arrays were washed and scanned with the PowerScanner (Tecan) and analyzed with Array-Pro Analyzer 6.3 software (MediaCybernetics). Array features were extracted with the Agilent’s Feature Extraction software (Agilent Technologies, CA, USA). A total of 16 arrays were hybridized and biological dye-swaps were performed for each sample. The raw and normalized data of 16 arrays have been deposited on the GEO public functional genomics data repository with GEO accession number GSE69173.

### Array data and downstream analysis

The array data and downstream analyses methods used for the study have been described in EmbryoGENE Microarray (EDMA) platform [25]. Briefly, the intensity cutoff (Mean + 4*SD) of the negative controls was used to identify probes that displayed a signal intensity above the background. Loess normalization followed by quantile inter-array scale normalization was performed to obtain Bayesian statistics of differential methylation. Differentially methylated probes were then identified from the full set of probes using linear models for microarray data (limma) [27]. Since in EDMA platform, the measured fold changes are not quantitative but merely indicative of higher or lower odds of differentially methylation, probes which showed significant (p < 0.05) differences with absolute log_2_(fold-change) ≥1.5 between the treatment and the reference sample were considered as differentially methylated regions (DMRs). Therefore, probes which showed a significant increase in signal intensity by 1.5 folds and higher in ZY, 4C, 16C or IVP compared to the VO group were considered as hypermethylated probes while probes which displayed a reduced signal intensity by 1.5 folds and higher in ZY, 4C, 16C or IVP compared to the VO group were considered as hypomethylated probes. Enrichment analysis for genome-scale DNA methylation data was performed using string of integrated scripts that sorts the genome data into CpG island density, CpG island length, CpG island distance, genomic location and types of repetitive elements. Moreover, the gene ontology enrichment analysis was performed for genes that were associated with differentially methylated regions. Probes were associated with the GO terms of a gene provided the probe fragment falls within the introns, exons or promoter of a particular gene. Following this, Go enrichment was assessed using the topGO bioconductor package (http://www.bioconductor.org/packages/release/bioc/html/topGO.html) and the weight01 algorithm defined by Alexa et al [28]. For comparative analysis of methylation profile and gene expression data, the genes absolute log_2_(fold-change) ≥1.5, p value < 0.05 and false discovery rate (FDR) < 0.3 were selected from our previous data [22]. The heatmaps showing the relationship between differentially methylated regions and differentially expressed genes were constructed using PermutMatrix [29].

### Validation of differentially methylated genomic regions using bisulfite sequencing

Twelve differentially methylated regions (DMRs) were selected to validate the array data independently using bisulfite sequencing. For this, primers (Table 1) were designed using MethPrimer (http://www.urogene.org/cgi-bin/methprimer/methprimer.cgi), an online tool for designing primers for amplification of bisulfite converted DNA. The genomic DNA in ZY, 4C, 16C, IVP and VO blastocyst groups was bisulfite converted using EZ DNA methylation direct kit (ZymoResearch) according to the manufacturer’s instruction. PCR amplification was performed in 25 μl volume containing 2 μl of bisulfite-converted DNA, 0.4 μl forward and reverses primers (10μm), 0.4 μ1 dNTP mixes (25mM), 0.2 μl Zymo Taq^™^ DNA polymerase (5u/μl) (ZymoResearch). The presence of PCR product was confirmed by loading 5 μl of the PCR product onto electrophoresis run on 2% w/v agarose gel stained with ethidium bromide at 120 V for 20 min. The PCR product was then purified using QIAquick PCR Purification (Qiagen) and cloned to pGEM^®^-T Easy Vector Systems (Promega, WI, USA) and transformed to *E*. *coli* competent cells. The bacterial culture was plated onto LB agar/ampicillin/IPTG/X-gal plate and incubated overnight at 37°C. Following this, 10–20 independent white colonies were selected and sequenced in GenomeLab™ GeXP Genetic Analysis System (Beckman Coulter). The bisulfite sequencing DNA methylation analysis software (BISMA) [30] was used to analyze the sequencing data. The average of methylation percentage at each CpG site in each experimental group was used for analysis.

**Table 1:** The list of primers used to validate DMRs in ZY, 4C, 16C and IVP blastocyst groups using bisulfite sequencing.

Blastocyst group/s	EDMA_ID	Primers (5’—3’)	bp	Ann. Temp (TD°C)
IVP	13_09053(CTSZ)	F TTTGGGTTTTAGAAGATTGGATTTA	163	60–55
		R CATTACCACCACACCCTCTACTAAT		
IVP	24_05279 (LAMA1)	F GGTGTTTAGGATTTATTTGGAAATT	100	60–55
		R ACATACTTCCTATCCACTACCTTAAATAT		
4C, 16C&IVP	18_16952 (SYT1)	F TTTTTTTTAAATATAGGGAGATT	223	60–55
		R ATAAAAACAACAAACACTAAAACC		
IVP &16C	4_15002 (PTPRN2)	F TTTAGATTGTTTTTGGTGAGGAAT	130	53–43
		R TTCTATAAAAAAAACTATCAACCAC		
IVP	25_06288 (HS3ST4)	F GGAAATTTTAAAGTTTTTTAGTTAATT	198	60–55
		R TTACTCTACCTACCTCAAACTCCTC		
4C & IVP	01_04641(GPR156)	F AGGTTATGGGAATTATGAATTTTTT	158	60–55
		R ATACTTAACCAACTCCCCAAATAAC		
IVP	09_03250 (GPR6)	F TTTTGGTTTTAGAGTGTAAGTTAGG	160	60–55
		R TTCTCCCATCTATTCCTAAAAATTC		
ZY	15_10150 (CRY2)	F GTTGTAGAGGGAAGGTTTTAGTTTT	188	55–45
		R AAAAAAACTACTCCTCAAACCAATC		
16C	21_01192 (IGF1R)	F GGTATAGGATTTAGGAGGTGGAATT	115	60–50
		R CCACTTAAAAAACTTCTCAATCAATC		
ZY	28_04337 (KCNMA1)	F GGTTAGATTTTGGGTAATTATTAAGT	207	55–45
		R ACCAAACATAAACTCATCCTTTTCT		
4C	19_11212 (FBF1)	F AGTTTTTGTGAGAAGTTGTTGAAAGTA	207	55–45
		R AAAACTTAAATAAAAAACCCATCCC		
IVP	19_05756 (KCTD11)	F TGTGATTAATGAGGATAGGGTAGAG	233	55–45
		R CTTCAAAATCAAAAAAAACAAAATC		

bp = PCR product length in base pairs, Ann. Temp = annealing temperature of the primers, TD = Touch down PCR. EDMA_ID refers to the identification ID of each EDMA probe. Each ID is preceded by EDMA_MET_. The genes associated with each EDMA probe ID is indicated in parenthesis.

### Confirmation of candidate genes using qPCR

In the current study, the DNA methylation data and previously published transcriptome profile were merged to get insight into the relationship between methylation pattern and the gene expression pattern. Although, the transcriptome array data was validated previoulsy, here we randomly selected 7 differentially expressed genes to confirm their expression pattern using the RNA samples isolated from the same samples whose DNA was used for methylation analysis. For this, the total RNAs isolated from each group of blastocysts were reverse transcribed using Universal cDNA synthesis kit (Exiqon) according to the manufactures recommendation. Briefly, a 20 μl reaction mix consisting of total RNA, reaction buffer, enzyme mixes and nuclease-free water was incubated at 42°C for 60 min, followed by 5 min incubation at 95°C. Gene specific primers (S2 Table) were designed and their PCR product specificity was confirmed sequencing the PCR products in GenomeLab™ GeXP Genetic Analysis System (Beckman Coulter). The qPCR was then performed in 20 μl reaction volume containing iTaq SYBR Green Supermix with ROX (Bio-Rad laboratories Germany), the cDNA samples of ZY, 4C, 16C, IVP or VO blastocyst group, the specific forward and reverse primer in the StepOnePlus™ Real-Time PCR Systems (Applied Biosystems, Foster city, CA). The qPCR thermal cycling parameter was set as 95°C for 3 min followed by 40 cycles of 95°C for 15 s and 60°C for 1 min. Following this, dissociation curve was generated by starting fluorescence acquisition at 60°C and measurements were taken every 7 s interval until the temperature was reached 95°C. The qPCR was performed in four replicates and analyzed using comparative (Ct (2^−ΔΔCt^)) method. The specificity of amplification was evaluated by monitoring the dissociation (melting) curve of each candidate gene. Glyceraldehyd-3-phosphate-dehydrogenase (GAPDH) was used as endogenous normalizer.

## Results

### Differentially methylated genomic regions in blastocysts developed from embryos exposed to *in vitro* culture in stage specific manner

**Fig 1:**
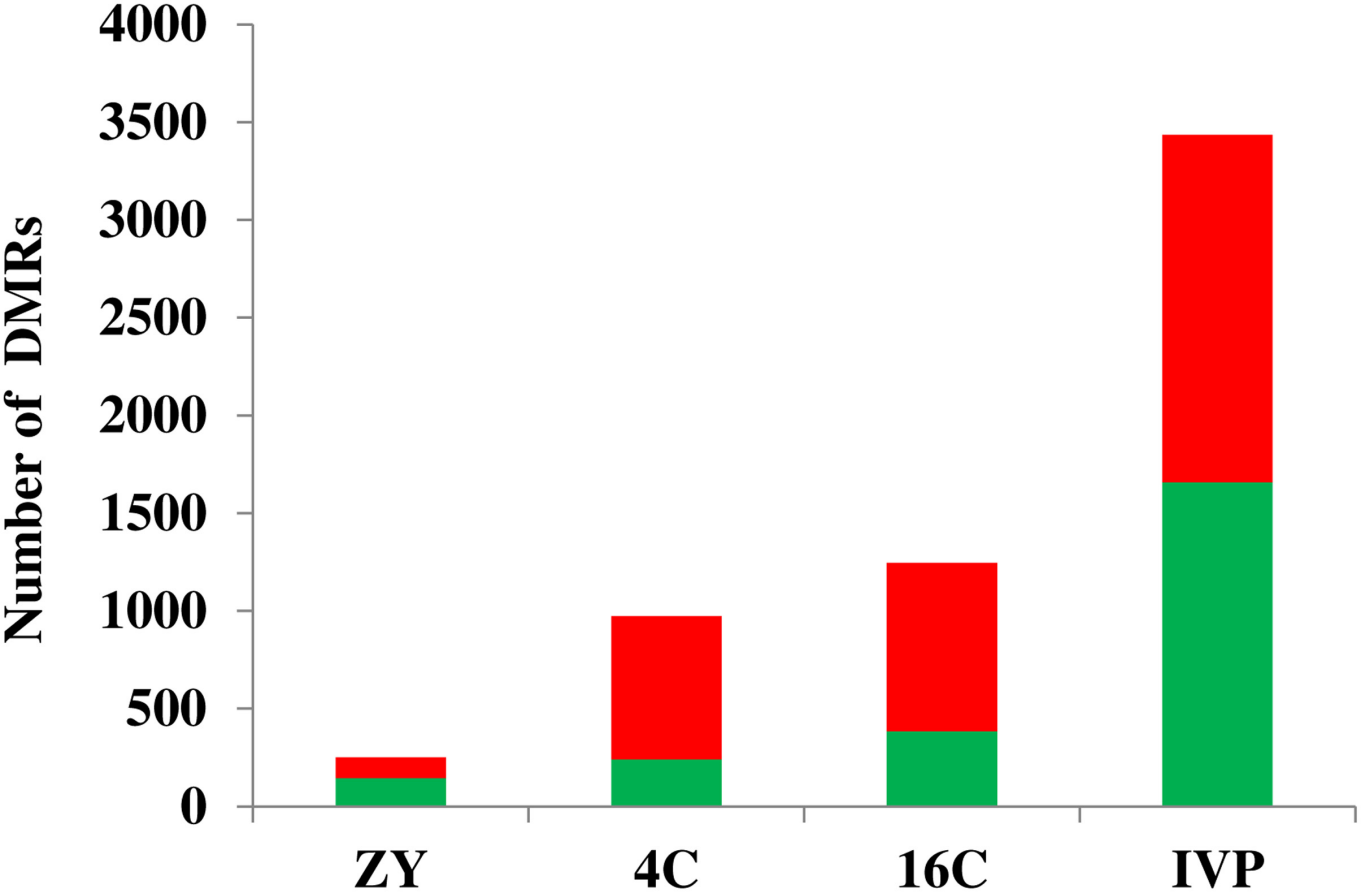
Differentially methylated regions (DMRs) in ZY, 4C, 16C and IVP blastocyst groups. Red and green bars indicate the number of hypermethylated and hypomethylated genomic regions, respectively. The magnitude the DMRs stretched between 1.5 and 2.3 absolute fold changes in ZY and 4C groups, between 1.5 and 4.6 in the 16C group and between 1.5 and 2.6 folds in IVP group with reference to VO group.

**Fig 2:**
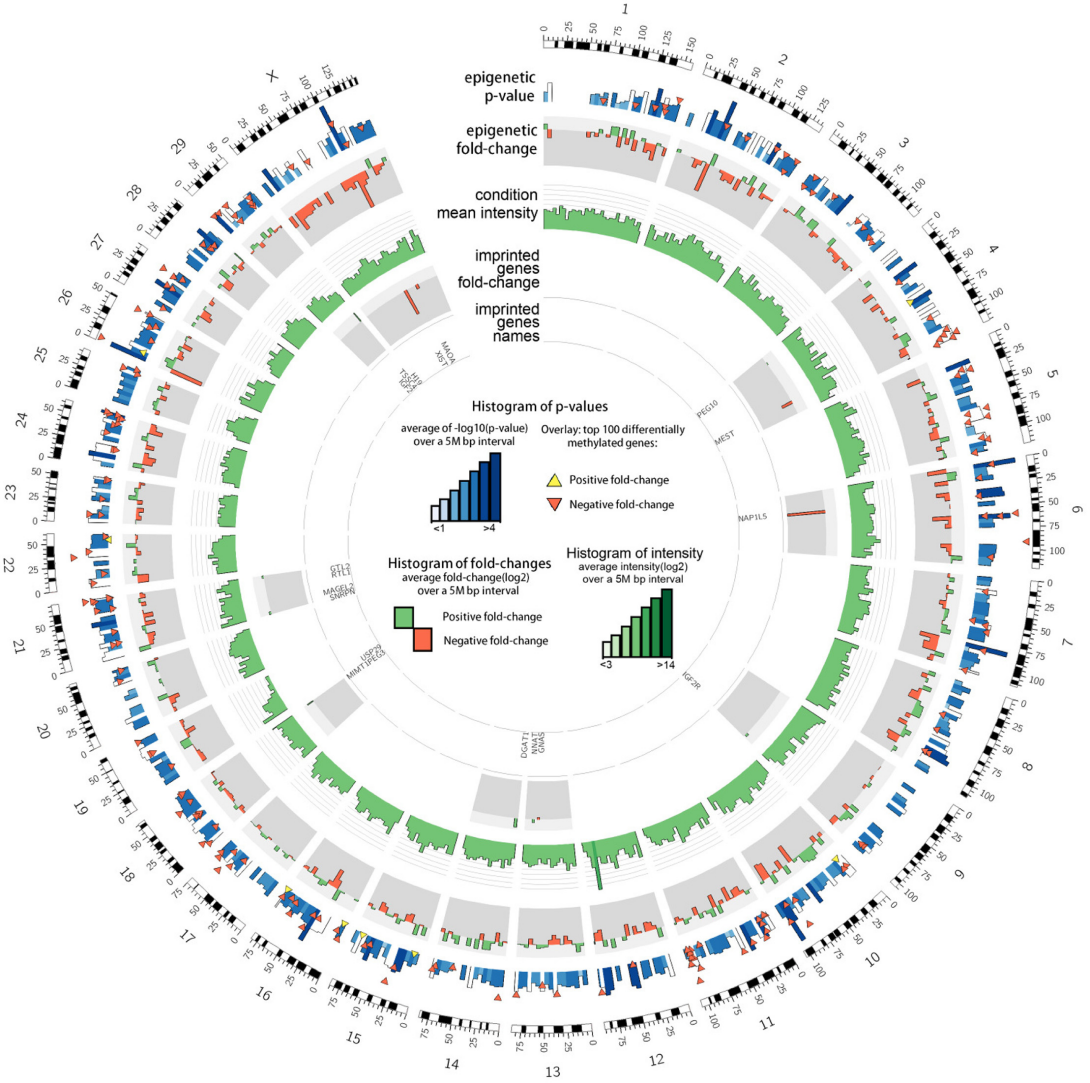
The circos plot representing the overall methylation levels in the IVP blastocyst group. The mean p-values of 5 Mbp windows are indicated along with the 100 most significant DMRs. Positive and negative fold-changes represent the level of hypermethylation and hypomethylation in IVP blastocysts.

In this study, we investigated the DNA methylation landscape of blastocysts developed in vivo after embryos were cultured in vitro until zygote (ZY), 4-cell (4C) or 16-cell (16C) stage and blastocysts developed completely under in vitro condition (IVP) with reference to the blastocysts developed completely under in vivo condition (VO). For this, the blastocysts genomic DNA was digested using the MseI restriction enzyme followed by adapter ligation, methyl-sensitive restriction enzymes and ligation mediated PCR genomic amplification. The abundance of each fragment obtained from ligation mediated amplification in each sample was then measured using EmbryoGENE DNA Methylation Array (EDMA). Probes with signal intensities higher than the background signal in each comparison (ZY vs. VO, 4C vs. VO, 16C vs. VO and IVP vs. VO) were analyzed for further analysis. Accordingly, from a total of > 400K probes, the signal intensity of 196871, 199265, 255254, 248453 and 227075 probes in ZY, 4C, 16C, IVP and VO groups, respectively exceeded the background signal and 174460 probes were commonly detected in all sample groups (S1 Fig). Differentially methylated regions were identified by fitting intensity data using a linear model [27]. Probes that exhibited fold-changes of ≥ log_2_1.5 at p < 0.05 in each comparison were considered differentially methylated regions (DMRs). Thus, differential methylated region analysis indicated that longer exposure of preimplantation embryos to the in vitro culture conditions increased DNA methylation profile alteration in the resulting blastocysts (Fig 1). A systematic increase in both hypermethylated and hypomethylated genomic regions were identified from the ZY to the IVP group, with hypermethylated loci outpacing that of the hypomethylated ones in 4C and 16C blastocyst groups. Furthermore, 42, 75, 69 and 51.8% of the total DMRs in the ZY, 4C, 16C and IVP blastocyst groups, respectively were hypermethylated and the rest were hypomethylated. Apart from these, the DNA methylation profiles identified in ZY, 4C, 16C and IVP blastocyst groups were evenly distributed across all chromosomes without showing any significant chromosome specific effects (Fig 2, S2–S4 Figs).

### Stably hypermethylated or hypomethylated genomic loci

**Fig 3:**
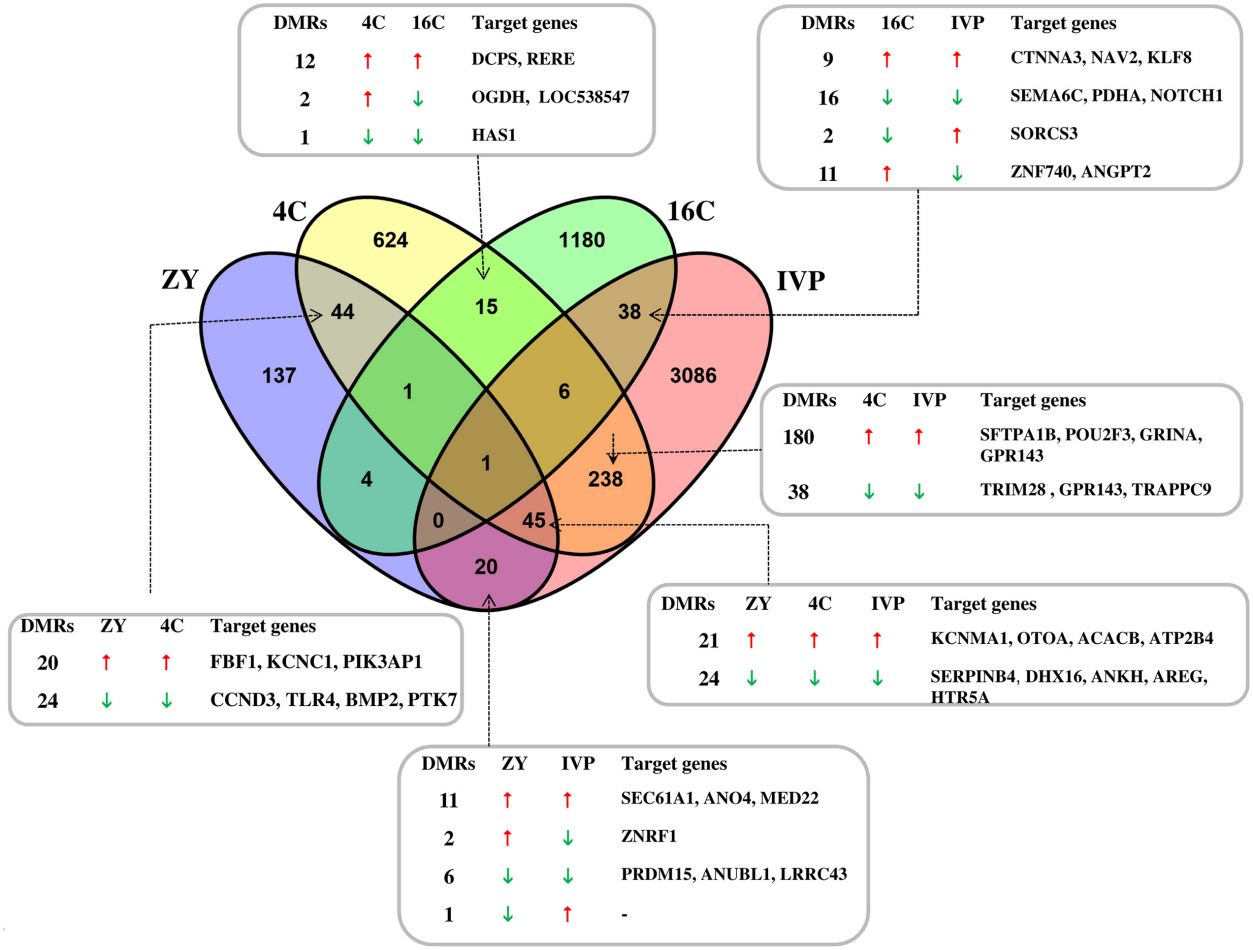
Venn diagram depicting differentially methylated genomic regions specific in ZY, 4C, 16C or IVP blastocyst group or genomic regions stably hypermethylated or hypomethylated in two or more blastocyst groups. Symbols, ↑ and ↓ indicate hypermethylation and hypomethylation, respectively. DMRs = the number of differentially methylated regions. The gene lists under the column target genes indicate only representative ones.

In order to get insight into the genomic loci that were differentially methylated in a stable or stage dependent manner, we looked into the DMRs that were specific to each blastocyst group and the DMRs that were common in two or more blastocyst groups. Hypermethylated or hypomethylated genomic loci which appeared in two or more blastocyst groups were considered as “stably” differentially methylated regions. Accordingly, a total of 137, 624, 1180, and 3086 DMRs were found to be specific to only ZY, 4C, 16C and IVP blastocyst groups, respectively, whereas 115, 350, 65 and 348 DMRs in ZY, 4C, 16C and IVP blastocyst groups, respectively were stably differentially methylated in two or more blastocyst groups (Fig 3). Moreover, differentially methylated genomic loci specific to each group were increased depending on stages of the embryos while stably differentially methylated genomic loci didn’t exhibit that trend. Among stably hypermethylated or hypomethylated genomic loci, 45 DMRs were identified in ZY, 4C and IVP groups, but these genomic loci were not significantly differentially methylated in the 16C blastocyst group (Fig 3, S3 Table). Furthermore, we look into a stage wise comparisons from ZY to IVP, it was evidenced that the ZY blastocyst group had relatively higher DMRs in common with 4C group than it did with other blastocyst groups (Fig 3, S3 Table). On the other hand, the 4C blastocysts shared relatively higher number of DMRs (n = 290; 29.7% of the total) with IVP blastocyst group and thus compared to other groups, in terms of common DMRs, the 4C blastocyst group was quite closer to the IVP blastocyst group (Fig 3, S5 Table) followed by ZY group. However, the 16C blastocyst group, which the EGA is believed to occur in vitro, shared only 3% of the DMRs (45/1245) with IVP blastocyst group (Fig 3).

#### Differentially methylated CpG islands in blastocysts of different groups

In EDMA platform, the CpG islands covered about 13% of the total probes. We used this opportunity to analyze differentially methylated CpG islands in each blastocyst group. The result has shown that the methylation pattern of CpG islands was found to be higher in IVP (n = 357) compared to ZY (n = 14), 4C (n = 35) and the 16C (n = 22) blastocyst groups. Furthermore, the data also evidenced that 42, 86, 71, and 50% of the differential methylated CpG islands in ZY, 4C, 16C and IVP groups, respectively were found to be hypermethylated. In addition, we also looked into whether the methylation patterns of CpG islands that fall within the promoter, exon or intron genomic region could differ between the blastocyst groups. Indeed, the genomic localization of these genomic loci indicated that in ZY group, differentially methylated CpG islands were more frequently at intron (Table 2) whereas in the 4C group, differentially methylated CpG islands were more frequently detected at exon (Table 3). Furthermore, in the 16C blastocyst group, the frequency of differentially methylated CpG islands was found to be similar in exonic and intronic regions (Table 4). Similarly, in IVP groups, the proportion of differentially methylated CpG islands detected on exonic region exceeded those located at the intron and promoter regions (S6 Table) although a total of 28 differentially methylated CpG islands were identified in promoter and proximal promoter regions (Table 5).

**Table 2:** Differentially methylated CpG islands in ZY blastocyst group.

EDMA_ ID	Chromosomal location of the DMRs								
	Chr. No	Start of the fragment	End of the fragment	Log_2_FC	P. value	CpG-I	CpG-L	Genes containing DMRs	Genomic region of DMRs
11_18575	11	105594543	105594899	0.69	0.0001	295	1310	MRPL41	Ppromoter
05_14532	5	111575190	111577414	-0.71	0.0229	578	285	CACNA1I-8	Exon
13_11174	13	65993807	65994316	0.69	0.0001	177	513	EPB41L1-15	Intron
05_07596	5	65012493	65013029	0.71	0.0047	320	292	ANO4-1	Intron
04_02015	4	17518725	17519010	0.73	0.0264	285	302	NXPH1-2	Intron
07_09727	7	45330243	45331467	-0.87	0.0120	1188	1364	MIDN-1	Exon
21_12449	21	70709771	70711215	-0.66	0.0040	120	1013	LOC615365-1	Intron
17_06810	17	60198116-	60199138	-0.79	0.0176	509	391	FBXO21-1-9	Intron

CpG_L is the length of the island on the genome while CpG- I indicates the number of bases within the fragment which are part of the CpG island. Numbers preceded by hyphen following the gene name indicate the exon or intron numbers depending whether the DMR is located in exon or intron of the gene. EDMA_ID refers to the identification ID of each EDMA probe. Each ID is preceded by EDMA_MET. Ppromoter = proximal promoter.

**Table 3:** Differentially methylated CpG islands in 4C blastocyst group.

EDMA_ ID	Chromosomal location of the DMRs								
	Chr. No	Start of the fragment	End of the fragment	Log_2_FC	P. value	CpG-I	CpG-L	Genes containing DMRs	Genomic region of DMRs
23_10636	23	50703682	50704205	0.717	0.0000	523	597	WRNIP1-1	Exon
14_00311	14	2021102	2022310	0.67	0.0023	1018	316	GRINA-7	Exon
07_01370	7	4445833	4448462	0.65	0.0002	1279	618	CRTC1-14,-15	Exon
29_08515	29	48125388	48126127	0.61	0.0038	624	217	PPFIA1-14	Exon
22_06844	22	47721645	47722113	0.59	0.0045	468	239	CACNA1D-	Exon
24_05434	24	41196455	41196870	0.90	0.0001	161	212	PTPRM-13	Intron
01_04641	1	65593765	65594620	0.78	0.0000	222	222	GPR156-7	Intron
12_00244	12	5198652	5199215	0.75	0.0001	563	2923	PCDH17-4	Exon
09_10794	9	103164941	103165323	0.71	0.0002	307	266	RPS6KA2-20	Intron
17_10379	17	70755932	70756476	0.64	0.0005	217	217	AP1B1-16	Exon
05_19144	5	121066466	121067409	0.63	0.0001	650	264	MLC1-1	Intron
24_08819	24	58000202	58000701	0.62	0.0005	207	207	NEDD4L-15	Intron
12_11235	12	90547550	90548313	0.61	0.0035	640	366	PCID2-3	Exon
09_09020	9	96614497	96615244	0.60	0.0006	719	248	EZR-8	Exon
26_08607	26	49756976	49757126	0.60	0.0013	78	234	GLRX3-4	Exon
08_12515	8	102986219	102986674	-0.64	0.0441	455	496	UGCG	Ppromoter
19_06047	19	28409208	28410192	-0.67	0.0054	134	947	VAMP2-2-4	Exon
18_16952	18	62701913	62702419	-0.69	0.0275	506	1529	SYT5-2	Exon
26_00146	26	4831864	4832162	-0.69	0.0187	65	327	PCDH1-1,-2	Intron
07_09727	7	45330243	45331467	-0.82	0.0282	1188	1364	MIDN-1	Exon
17_12362	17	74231699	74233071	0.72	0.0004	326	1318	PI4KA-37	Exon
08_00160	8	880729	882384	0.67	0.0013	1258	1258	PALLD-10	Intron
06_12635	6	118964269	118964779	0.63	0.0021	510	468	AFAP1-6	Intron
18_02124	18	8100425	8100718	0.62	0.0007	106	323	LOC782414-9	Intron
29_04592	29	36902489	36903114	0.60	0.0002	168	235	APLP2-15	Exon
05_19063	5	120935146	120936857	-0.61	0.0008	1711	6736	CRELD2-4-5	Exon

CpG_L is the length of the island on the genome while CpG- I indicates the number of bases within the fragment which are part of the CpG Island. Numbers preceded by hyphen following the gene name in the “Genes containing DMRs” column indicate the exon or intron number depending whether the DMR is located in exon or intron of the gene. EDMA_ID refers to the identification ID of each EDMA probe. Each ID is preceded by EDMA_MET. PPromoter. = proximal promoter.

**Table 4:** Differentially methylated CpG islands in 16C blastocyst group.

EDMA_ID	Chromosomal location of the DMRs								
	Chr. No	Start of the fragment	End of the fragment	Log_2_ FC	P. value	CpG-I	CpG-L	Genes containing the DMRs	Genomic region of DMRs
05_02664	5	26984478	26985074	0.95	0.0280	264	883	ZNF740-7	Exon
05_02793	5	27362031	27363304	0.70	0.0095	222	222	KERIA-1	Exon
14_04905	14	25007734	25008796	0.62	0.0147	1028	514	PLAG1-1	Exon
16_00691	16	2836340	2837002	0.74	0.0484	653	703	TMCC2-1	Exon
18_16952	18	62701913	62702419	1.76	0.0095	506	1529	SYT5-2	Exon
18_17305	18	63672206	63673560	0.69	0.0238	1354	1139	NLRP5-3	Exon
25_06288	25	23550537	23551982	-0.66	0.0183	866	723	LOC782286-1	Exon
15_01221	15	18449268	18449610	0.65	0.0144	291	905	KDELC2-7	Intron
15_01222	15	18449268	18449610	0.79	0.0128	291	905	KDELC2-7	Intron
01_10044	1	118475087	118475364	0.99	0.0004	277	834	SELT-5	Intron
03_14948	3	110252770	110254313	0.59	0.0230	493	1370	MAP7D1-17	Intron
11_14277	11	97170314	97170914	-0.77	0.0355	600	382	FAM125B-2	Intron
04_02959	4	31137285	31137718	0.69	0.0184	433	2997	RAPGEF5-19	Intron
19_00766	19	8526575	8526970	0.60	0.0204	167	262	MSI2-5	Intron
19_00767	19	8526575	8526970	0.65	0.0141	167	262	MSI2-5	Intron
27_00593	27	4507450	4507765	0.68	0.0101	315	247	ANGPT2- 1–8	Intron
25_00821	25	1241931	1242218	0.72	0.0020	287	2812	CRAMP1L	Promoter
07_09707	7	45298929	45299941	-0.63	0.0174	1012	4231	ATP5D	Promoter
22_11951	22	59732686	59733347	1.10	0.0149	420	1492	ISY1	Ppromoter

CpG_L is the length of the island on the genome while CpG- I indicates the number of bases within the fragment which are part of the CpG Island. Numbers preceded by hyphen following the gene name in the “Genes containing DMRs” column indicate the exon or intron number depending whether the DMR is located in exon or intron of the gene. EDMA_ID refers to the identification ID of each EDMA probe. Each ID is preceded by EDMA_MET. Ppromoter = proximal promoter.

**Table 5:** Differentially methylated CpG islands at the promoter and proximal promoter regions in IVP blastocyst group.

EDMA_ID	Chromosomal location of DMRs								
	Chr. No	Start of the fragment	End of the fragment	Log_2_ FC	P value	CpG-I	CpG_L	Genes containing DMRs	Genomic region of DMRs
29_00040	29	645376	646080	0.84	0.0003	363	1200	*PANX1*	Ppromoter
29_04685	29	37141651	37143104	0.71	0.0127	310	1924	*ADAMTS15*	ppromoter
29_02699	29	25474801	25475647	0.64	0.0002	846	1918	*NAV2*	Ppromoter
05_13915	5	109960314	109960564	-0.63	0.0253	250	533	*GGA1*	Ppromoter
05_07794	5	67040732	67041118	-0.63	0.0035	177	237	*PAH*	Ppromoter
25_06093	25	22177033	22177350	-0.65	0.0021	260	584	*CACNG3*	Ppromoter
09_02104	9	28828997	28829168	-0.69	0.0071	171	910	*SMPDL3A*	Ppromoter
12_06617	12	62665617	62665887	-0.80	0.0000	178	336	*SLITRK5*	Ppromoter
29_06339	29	44062208	44063331	-0.84	0.0002	860	975	*CAPN1*	Ppromoter
09_03387	9	41554357	41555369	-0.86	0.0035	1012	832	*LOC519522*	Ppromoter
04_01262	4	10724605	10726568	-0.93	0.0005	302	286	*CALCR*	Ppromoter
16_12980	16	78787478	78788408	-0.98	0.0011	930	10103	*LHX9*	Ppromoter
02_17477	2	136185489	136187433	-1.04	0.0001	1143	879	*MFAP2*	Ppromoter
27_04888	27	37017432	7017742	-1.30	0.0000	135	218	*SLC20A2*	Ppromoter
17_12743	17	74699937	74701040	0.94	0.0002	353	1049	*HIRA*	Promoter
07_09681	7	45249958	45251190	0.84	0.0011	1172	1050	*SBNO2*	Promoter
18_03189	18	11124182	11124888	0.78	0.0224	512	353	*ZDHHC7*	Promoter
02_15404	2	132517660	132518606	0.73	0.0053	946	880	*SH2D5*	Promoter
25_01092	25	1698431	1700218	0.71	0.0001	275	275	*TRAF7*	Promoter
18_13165	18	52565414	52566378	0.64	0.0091	551	337	*ZNF235*	Promoter
13_07039	13	47917515	47918741	0.61	0.0210	1117	1102	*PROKR2*	Promoter
25_00091	25	209893	211620	-0.62	0.0000	969	898	*HBA*, *HBQ1*	Promoter
21_02630	21	19980747	19981499	-0.63	0.0011	312	1488	*MIR1179*, *MIR7*	Promoter
29_05558	29	41934496	41934798	-0.85	0.0269	302	1146	*CHRM1*	Promoter
11_16905	11	103077174	103077814	-0.88	0.0072	548	374	*GTF3C5*	Promoter
25_06124	25:	22457928	22458280	-1.19	0.0002	352	1146	*RBBP6*	Promoter
14_01401	14	4125691	4126327	-1.64	0.0001	459	294	*EIF2C2*	Promoter
03_01492	3	12257587	12258492	-2.04	0.0000	522	276	*LOC781123*	Promoter

CpG_L is the length of the island on the genome while CpG- I indicates the number of bases within the fragment which are part of the CpG Islands. EDMA_ID refers to the identification ID of each EDMA probe. Each ID is preceded by EDMA_MET. Ppromoter = proximal promoter.

#### Characterization of the differentially methylated CpG islands with respect to their length

**Fig 4:**
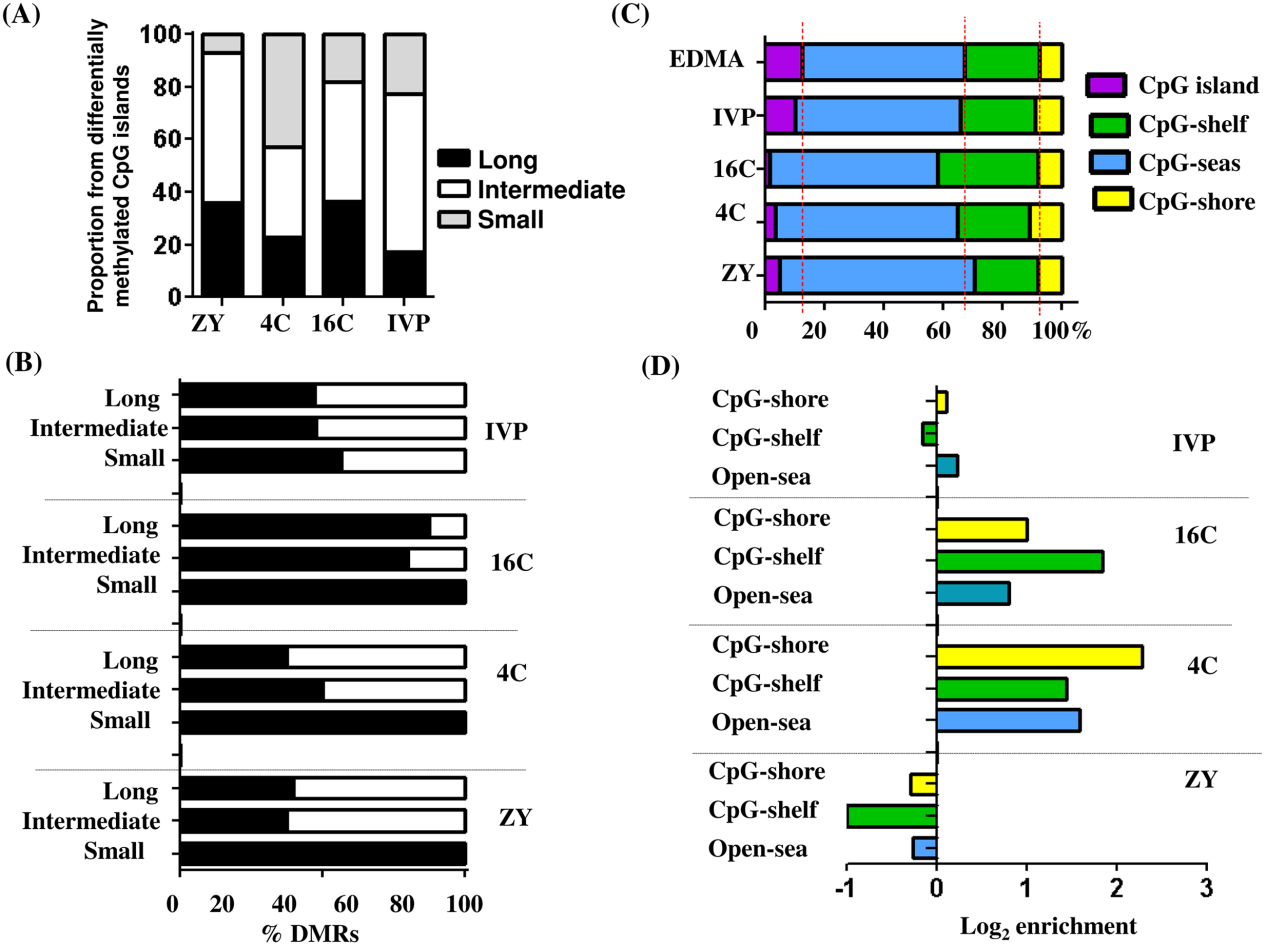
Differentially methylated CpG islands in ZY, 4C, 16C and IVP blastocyst groups. **(A), The relative proportion of long, short and intermediate CpG islands with respect to the total differentially methylated CpG islands. (B), The relative proportion of hypermethylated or hypomethylated small, long and intermediate CpG islands in ZY, 4C, 16C and IVP blastocyst groups.** Black and white bars indicate hypermethylation and hypomethylation, respectively. **(C), The relative proportion of CpG islands and non-CpG islands with respect to the total probeset (EDMA array) or relative to the total differentially methylated probes.** The red dotted line represents the baseline of the ratio when all probes are taken into account. The probe is assumed to be in CpG shore, CpG shelves and Open Sea if the fragment the probe is located 1–2 kbp, 2–4 kbp and > 4 kbp away from the nearest CpG island, respectively. **(D), Enrichment ratios of hypermethylated non-CpG islands DMRs with respect to their distance to the nearest CpG islands.** Positive log_2_ enrichment indicates increased proportion of hypermethylated region and negative log_2_ enrichment values depict higher proportion of hypomethylated genomic regions at Open-Sea, CpG-shore or CpG-shelf.

In the EDMA probes, the lengths of CpG islands were partitioned as a function of percentiles in which the bottom 20 and the top 80 percentiles were classified as short and long CpG islands, respectively and the CpG islands between the bottom 20 and the top 80 percentiles were classified as intermediate islands. Thus, using this option, we determined the methylation pattern of short, long and intermediate CpG islands in blastocysts of different groups. The result showed that considerable differences in proportion differentially methylated small, intermediate and long CpG islands were observed between the blastocyst groups (Fig 4A). The small CpG islands were hypermethylated in ZY, 4C and 16C groups, while long and intermediate-length CpG islands were hypermethylated in the 16C blastocyst group. However, in IVP group, as much hypomethylation as hypermethylation were detected in long or intermediate CpG islands (Fig 4B). Indeed, differentially methylated CpG islands covered only 2–10% of the total DMRs while 90–98% of the DMRs were located outside the CpG islands (Fig 4C). Therefore, the non-CpG island DMRs were classified into CpG shores, Open Sea and CpG shelf when the probe is located 1–2 kbp, 2–4 kbp and > 4 kbp away from the nearest CpG island, respectively. Thus, distribution of DMRs in CpG shore, Open Sea and CpG shelf tended to be different between the blastocyst groups (Fig 4C). Moreover, the proportion of differentially methylated region in CpG shore, Open Sea and CpG shelf in IVP blastocyst group exhibited a similar trend to that the proportion found in the whole array (EDMA). However, the expected proportion of DMRs in CpG shore, Open Sea and CpG shelf was deviated in ZY, 4C and 16C blastocyst groups compared to the proportion found in the whole array (EDMA) indicating the presence of selective differential methylation of genomic loci adjacent to the CpG islands depending on the stage of the embryos exposed to suboptimal culture condition (Fig 4C). Moreover, unlike to ZY and IVP groups, hypermethylated genomic loci were increased in CpG shore, Open Sea and CpG shelf in 4C and 16C blastocyst groups (Fig 4D).

#### Differentially methylated repetitive elements

**Fig 5:**
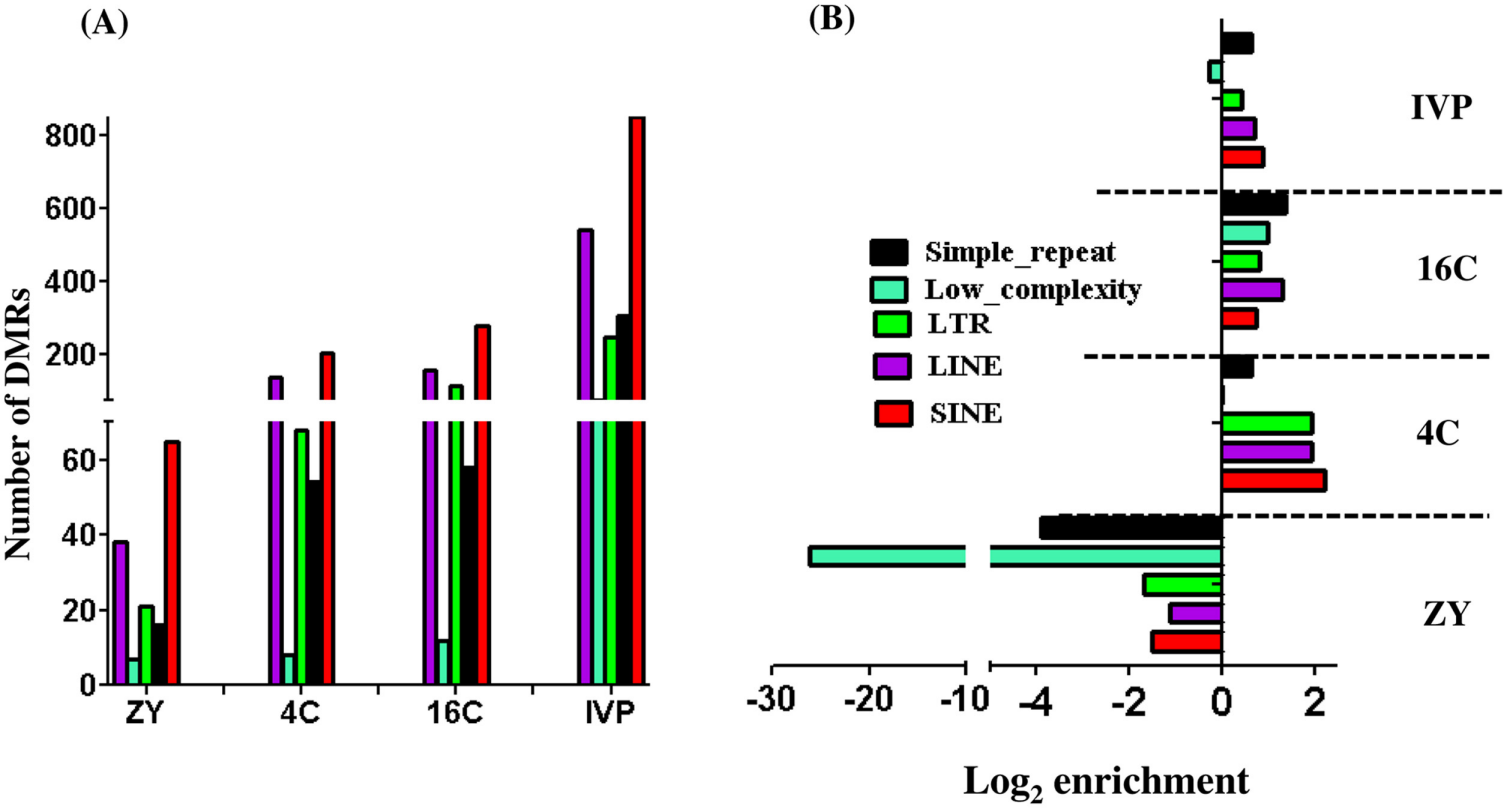
Differentially methylated bovine repetitive elements in different blastocyst groups. **(A), The number of differentially methylated long-terminal-repeat (LTR), short-interspersed repetitive elements (SINEs), long-interspersed repetitive elements (LINEs), low-complexity repetitive elements and simple repeats in ZY, 4C, 16C and IVP blastocyst groups. (B), Enrichment analysis indicating enrichment ratio of hypermethylated LTR, SINEs, LINEs, low-complexity repetitive elements or simple repeats of in ZY, 4C, 16C and IVP blastocyst groups.** Positive log_2_ enrichment values indicate higher proportion of hypermethylated LTR, SINEs, LINEs, low-complexity repetitive elements or simple repeats.

The repetitive elements, namely the long-terminal-repeat (LTR) retrotransposons, short-interspersed repetitive elements (SINEs), long-interspersed repetitive elements (LINEs), low-complexity repetitive elements and simple repeats were also differentially methylated in in ZY, 4C, 16C and IVP blastocyst groups. The number of differentially methylated SINE, LINE, LTR and low complexity repeats were relatively higher in IVP followed by 4C and 16C blastocyst groups (Fig 5A). Moreover, the majority of these classes of bovine repetitive elements tended to be hypomethylated in ZY and hypermethylated in 4C, 16C and IVP blastocyst groups (Fig 5B).

### Genomic localization of differentially methylated regions

**Fig 6:**
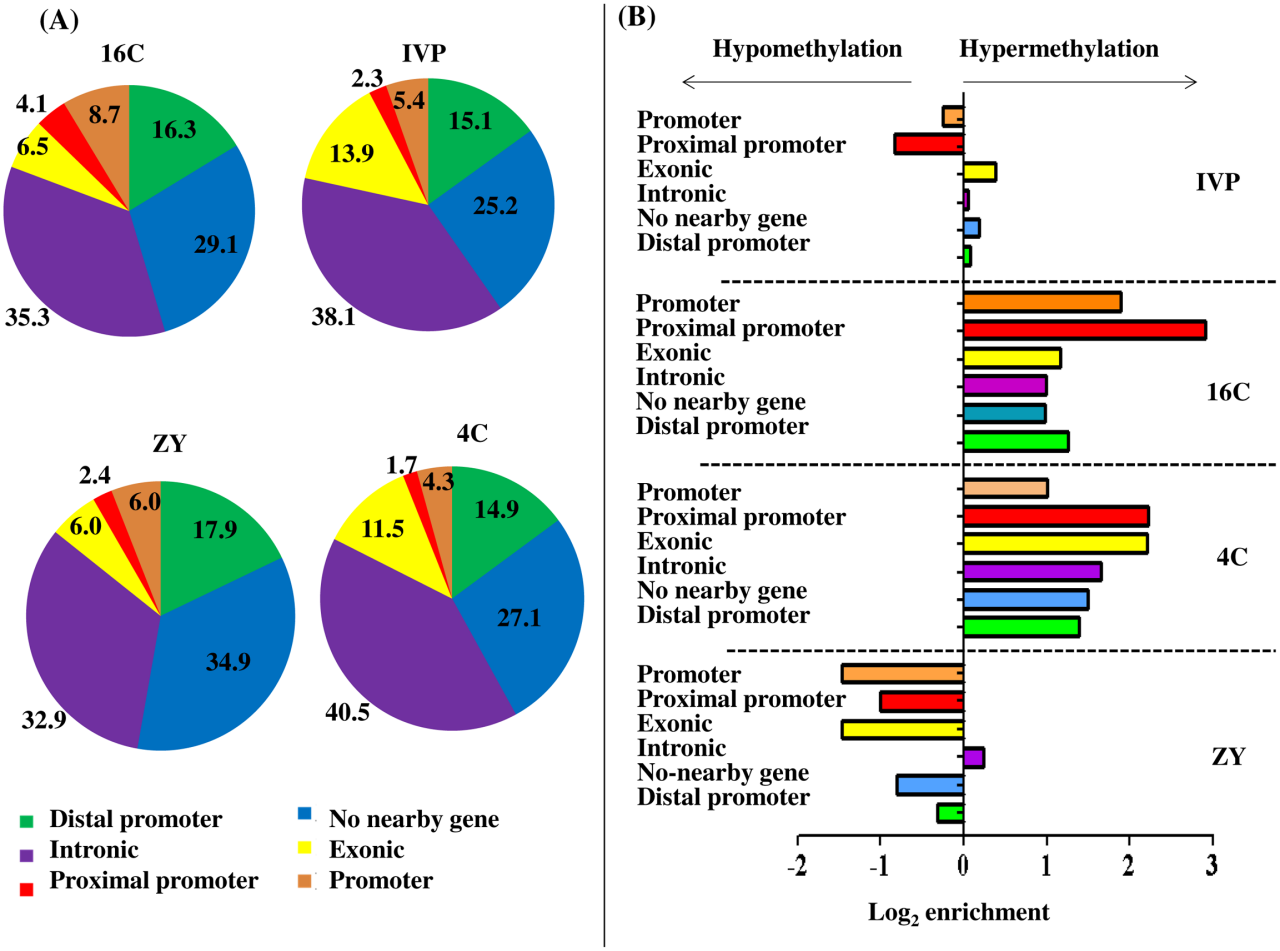
Genomic localization of differentially methylated regions. **(A), The proportion of DMRs falling within the promoter, proximal promoter, exonic, intronic or distal promoters in ZY, 4C, 16C and IVP blastocyst groups.** (**B), Enrichment ratio of hypermethylated regions in gene body and promoter sites in ZY, 4C, 16C and IVP blastocyst groups.** Positive log_2_ enrichment indicates higher proportion of the hypermethylated probes at the proximal promoter, promoter, distal promoter, intron or exon regions. The proximal promoter, promoter and distal promoter regions are defined as the first 1 kbp, 5 kbp and 50 kbp from the transcription start site, respectively and probes which are not within the "Distal promoter" distance were classified as "no nearby gene".

Apart from characterizing with respect to the CpG islands, we analyzed the distribution of all the DMRs with respect to their genomic position relative to the nearby genes. This analysis indicated that 65, 73, 71 and 75% of the DMRs in ZY, 4C, 16C and IVP, respectively were located in and around the promoter or gene body regions (Fig 6A). Moreover, genomic enrichment analysis revealed that in the ZY blastocyst group, the hypermethylated probes tended to be enriched only in the intronic regions whereas in the 4C and 16C groups, hypermethylated regions were enriched in intron, exon and promoter regions. On the other hand, in the IVP group, as much hypermethylation as hypomethylation were detected in intronic, exonic and promoter regions (Fig 6B).

### Functional annotation of genes affected by differentially methylated regions

**Fig 7:**
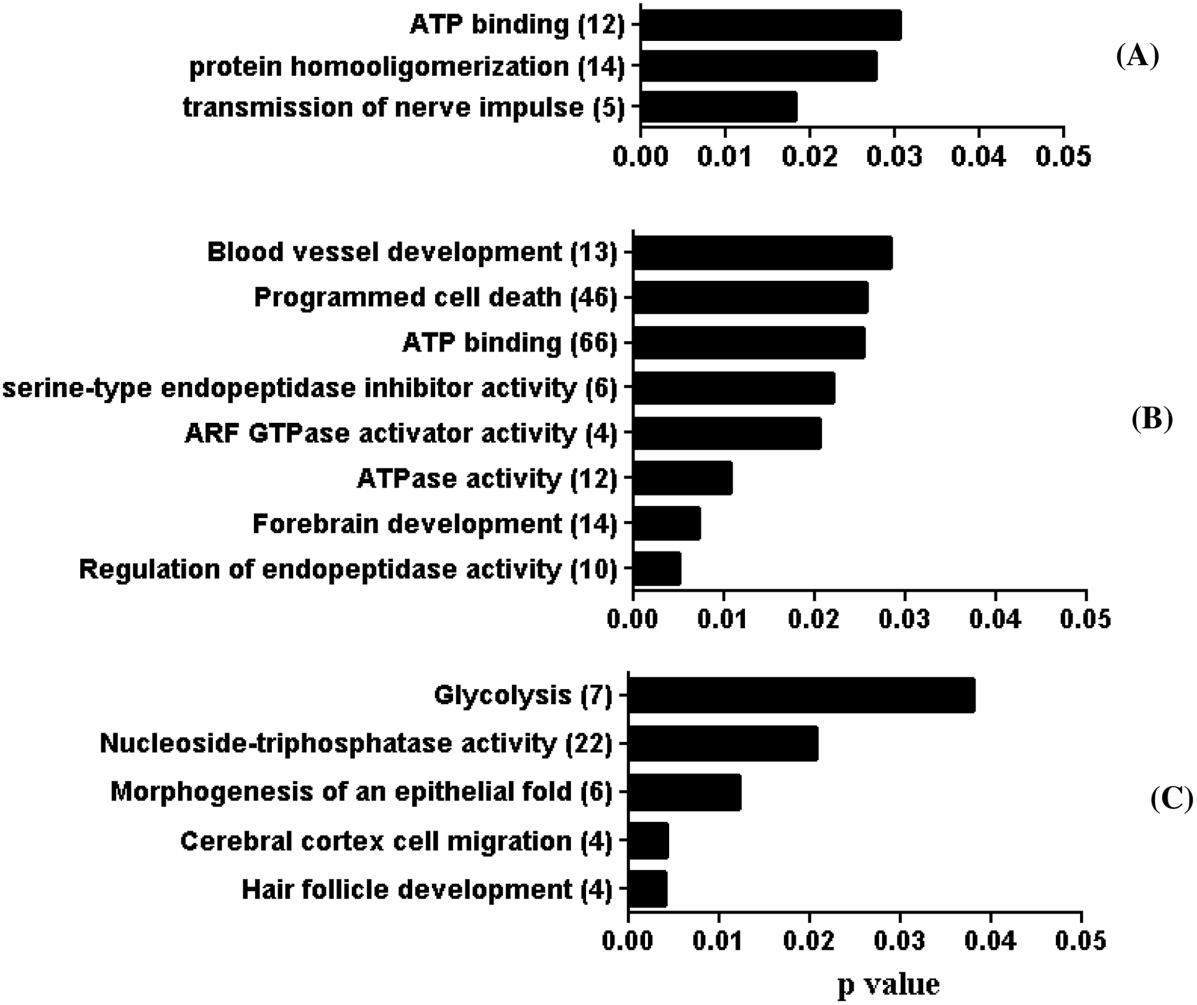
Functional annotation of genes targeted by differentially methylated region in ZY (A), 4C (B) and 16C (C) blastocyst groups. Numbers in parenthesis indicate the number of genes affected by DMRs.

**Fig 8:**
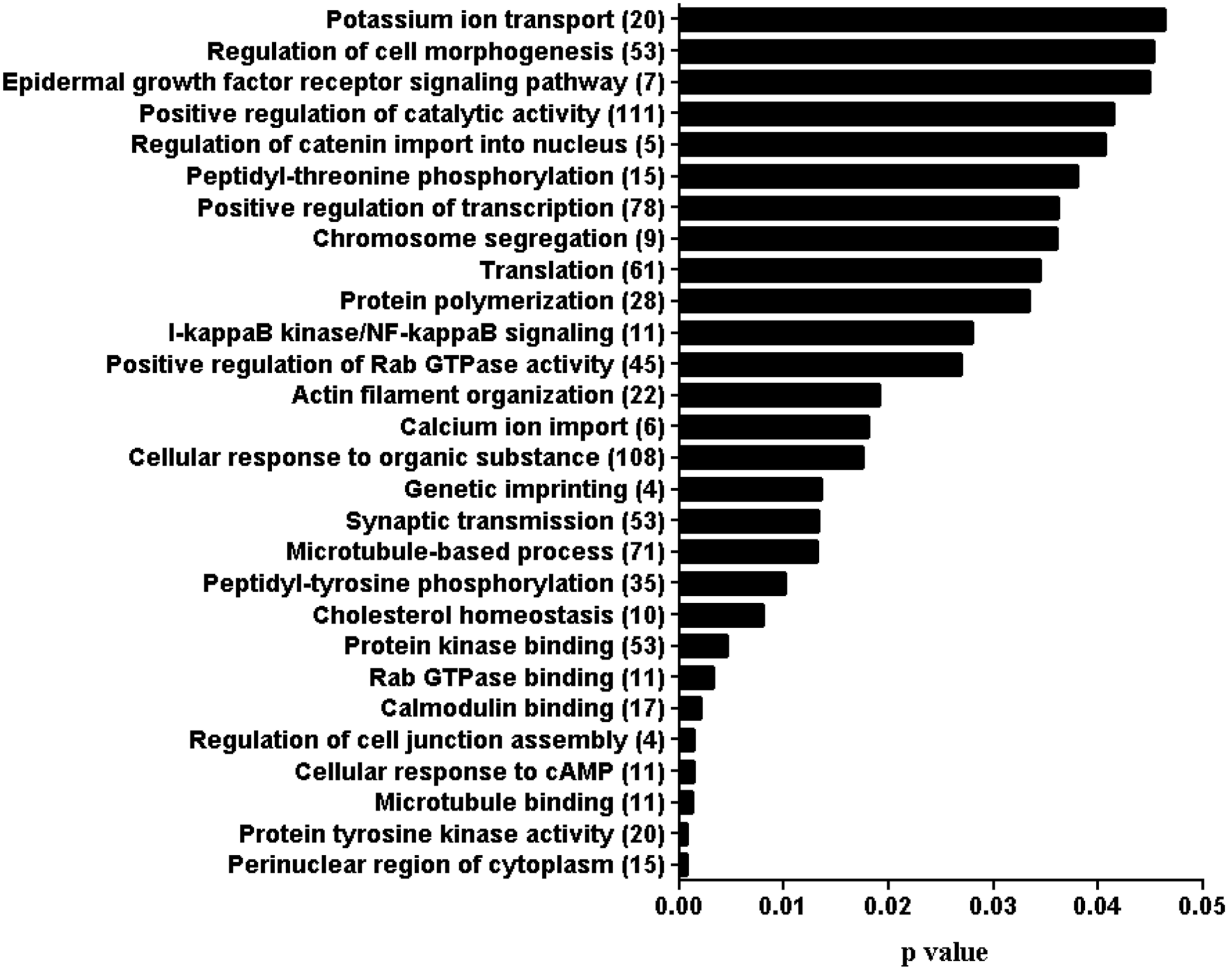
Functional annotation of genes targeted by differentially methylated regions in IVP blastocyst group. Numbers in parenthesis indicate the number of genes affected by the DMRs.

To get insight into the functional elements of the genomic region affected by the changes in methylation profile, gene ontology (GO) enrichment of the DMRs was performed using the topGO bioconductor package along with the algorithm defined by Alexa et al [28]. Probes which are located within the gene body or within 4 kbp of its transcription start site were considered for GO terms analysis. Accordingly, protein homooligomerization and ATP binding functional categories were affected in the ZY group (Fig 7A). On the other hand, at least 9 GO terms including ATP binding, programmed cell death and ATPase activity in the 4C blastocyst group (Fig 7B) and 4 gene ontology terms including glycolysis and nucleoside triphosphatase activity in the 16C blastocyst group (Fig 7C) were found to be affected by DMRs. In the IVP blastocyst group, the DMRs were associated with 83 gene ontology terms including genetic imprinting, regulation of transcription, metaphase/anaphase transition, chromosome segregation, cholesterol homeostasis and tricarboxylic acid cycle (Fig 8).

### Comparative analysis of DNA methylation and transcriptome profile

To determine whether the methylation profile changes in a given loci were associaited with changes in gene expression pattern, the DNA methylation patterns of ZY, 4C, 16C and IVP blastocyst groups were overlapped to our previous transcriptome data generated from the same blastocyst groups [22]. The analysis indicated that among the DMRs that had transcriptome profile information, 4.3% (n = 11) in ZY, 3.5% (n = 35) in 4C, 4.1% (n = 52) in 16C and 5.6% (n = 191) in IVP group were overlapped to the differentially expressed genes (DEGs) while the rest were associated with genes that were not significantly differentially expressed.

**Fig 9:**
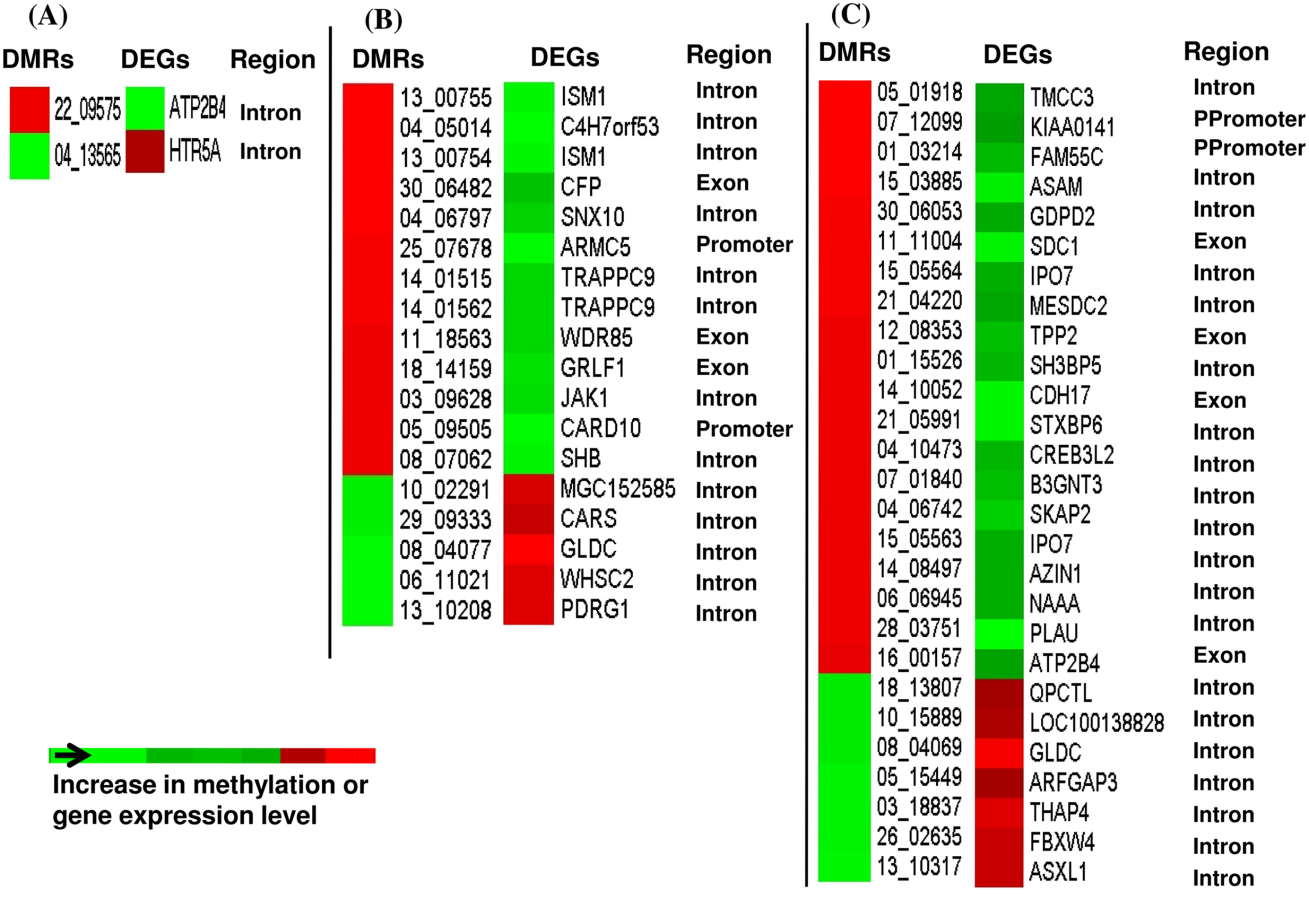
Heatmaps demonstrating inverse relationship between the patterns of gene expression and DNA methylation profile in ZY (A), 4C (B) and 16C (C) blastocyst groups. DMRs = differentially methylated regions, DEGs = differentially expressed genes, Region = Genomic region of the DMRs. Ppromoter = proximal promoter.

**Fig 10:**
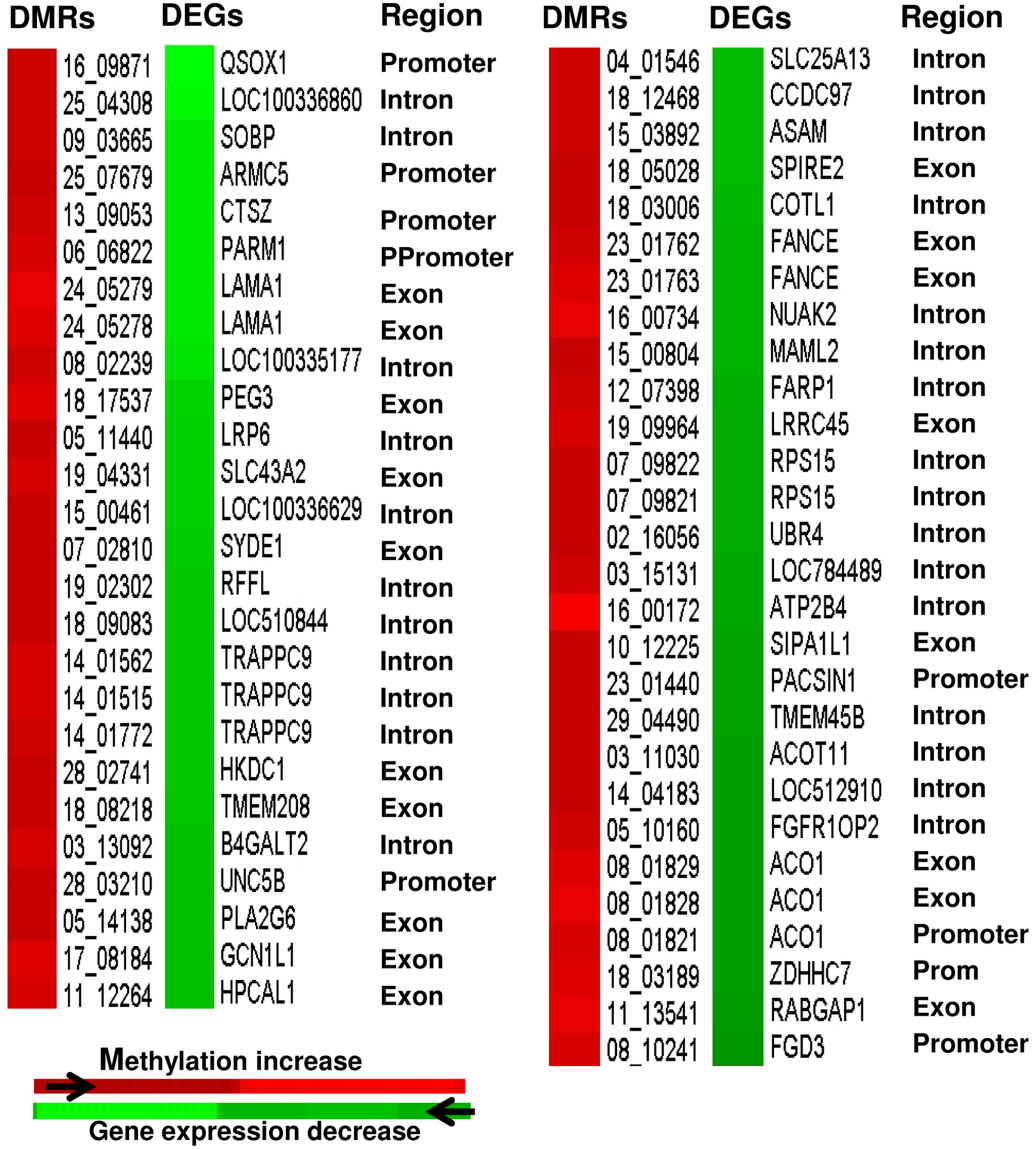
Heatmaps displaying inverse relationship between the hypermethylated genomic regions and downregulated gene expression patterns in IVP blastocyst group. Region = genomic Region of the DMRs. Ppromoter = proximal promoter.

**Fig 11:**
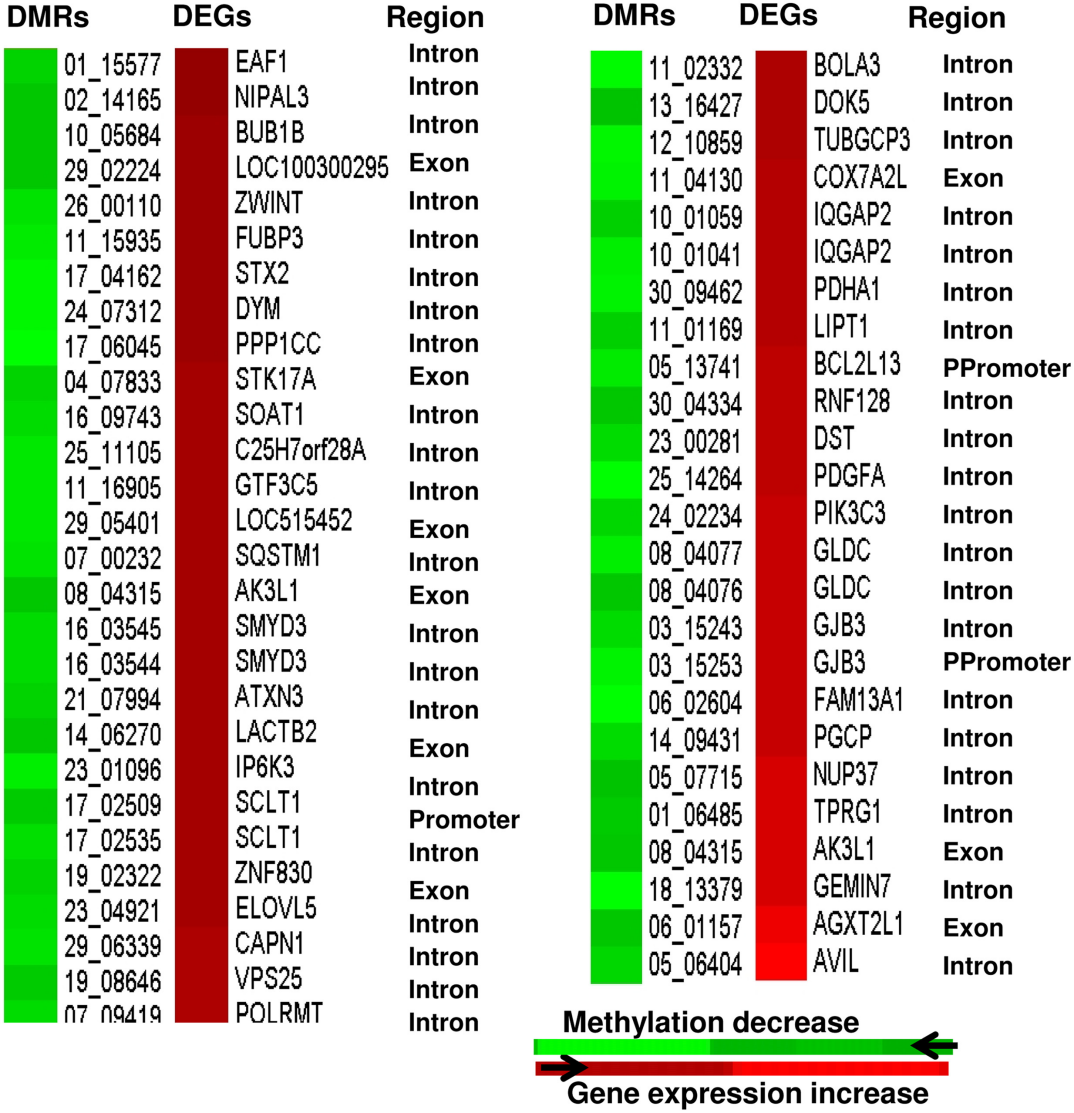
Heatmaps displaying inverse relationship between the hypomethylated genomic regions and increased expression pattern of their corresponding genes in IVP blastocyst group. DMRs = differentially methylated regions, DEGs = differentially expressed genes Region = genomic regions of the DMRs. Ppromoter = proximal promoter.

Considering that a higher methylation level should relate to a lower gene expression pattern [11], we opted to identify the DMRs that are negatively correlated with their corresponding gene expression pattern in each of the four blastocyst group. In ZY blastocyst group, although 11 DMRs were overlapped to 11 DEGs, only two DMRs (1.6% of the DMRs overlapped to the transcriptome data) exhibited inverse correlation (Fig 9A), while the methylation profile of 10 DMRs were positively correlated with the gene expression pattern (S5A Fig) at gene body and promoter regions. On the other hand, in the 4C blastocyst group, 19 DMRs (3.4% of the DMRs overlapped to the transcriptome data) including those located at *TRAPPC9*, *ISM1*, *GLDC* and *JAK1* genes were found to be inversely correlated with the gene expression profile (Fig 9B), while the methylation pattern of 17 DMRs was positively correlated with the gene expression (S5B Fig). Similar trend was observed in the 16C blastocyst group of which 3.9% of the total DMRs mapped to the transcriptome data were found to be inversely related to the expression pattern of their target genes (Fig 9C), but the mRNA levels of genes associated with 14 hypermethylated and 11 hypomethylated regions were positively correlated to the DNA methylation pattern (S5C Fig). In IVP blastocyst group, a total 107 DMRs (9.4% of the total DMRs mapped to the transcriptome data) comprising genes involved in cell death (*ATXN3*, *UNC5B*, *NUAK2*, *BCL2L13*, *FGD3*, *PEG3*) and cell division (*STX2*, *ZWINT*, *ZNF830*, *BUB1B*, *NUP37*, *PPP1CC*), displayed a negative correlation with the corresponding gene expression (Figs 10 and 11). However, the expression levels of genes associated with 24 hypermethylated genomic loci were increased and those associated with 60 hypomethylated DMRs were reduced compared to the in vivo blastocyst group (S6 Fig).

### Validation of differentially methylated region using bisulfite sequencing

**Fig 12:**
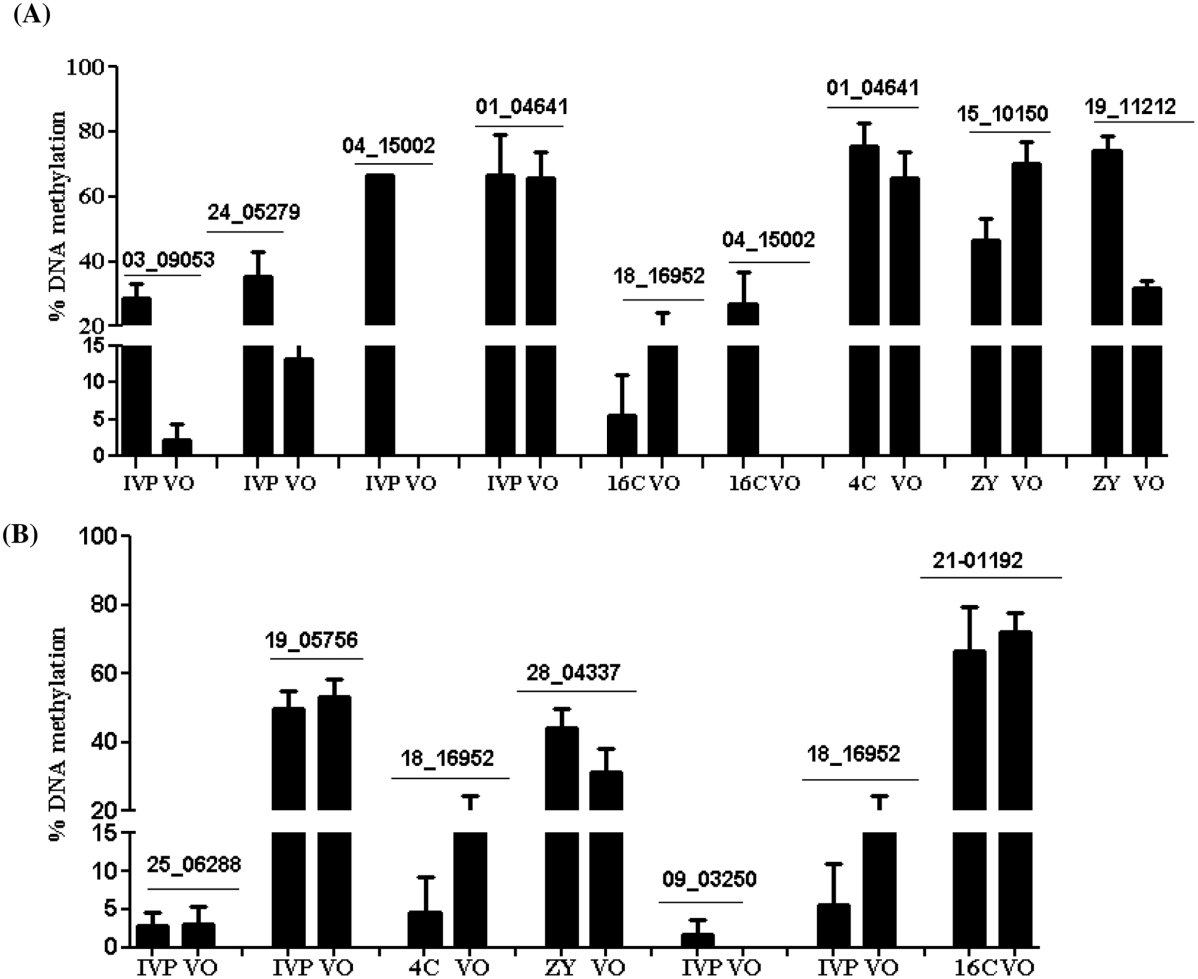
Validation of selected hypermethylated (A) and hypomethylated (B) genomic regions in ZY, 4C, 16C and IVP blastocyst groups using bisulfite sequencing. The numbers on the top of the bars correspond to the EDMA probe IDs. Bars are presented as mean ± SEM.

We selected 12 differentially methylated regions located either within or outside of CpG islands to confirm the array data using bisulfite sequencing. Three of the DMRs, namely EDMA_MET_18_16952, EDMA_MET_04_15002 and EDMA_MET_01_04641 associated with *SYT5*, *PTPRN2* and *GPR156* genes, respectively were commonly detected in two or more blastocyst groups. Among the probes analyzed, EDMA_MET_18_16952, which is a corresponding to the *SYT5* gene was found to be hypomethylated both in 4C and IVP compared to the VO group and the bisulfite sequencing results confirmed the methylation level of this locus to be lower in both 4C and IVP compared to the VO group. Similarly, both bisulfite sequencing and the array results confirmed a higher methylation level of the fragment corresponding to the EDMA_MET_04_15002 (*PTPRN2* gene) in 16C and IVP and lower methylation level of the genomic fragment that corresponds to the EDMA_MET_13_09053 probe (associated with *CTSZ* gene) in IVP compared to the VO group. Therefore, the results of the bisulfite sequencing were by and large similar to the array data (Fig 12, S7 Fig).

### Validation of differentially expressed genes using quantitative polymerase chain reaction (qPCR)

**Fig 13:**
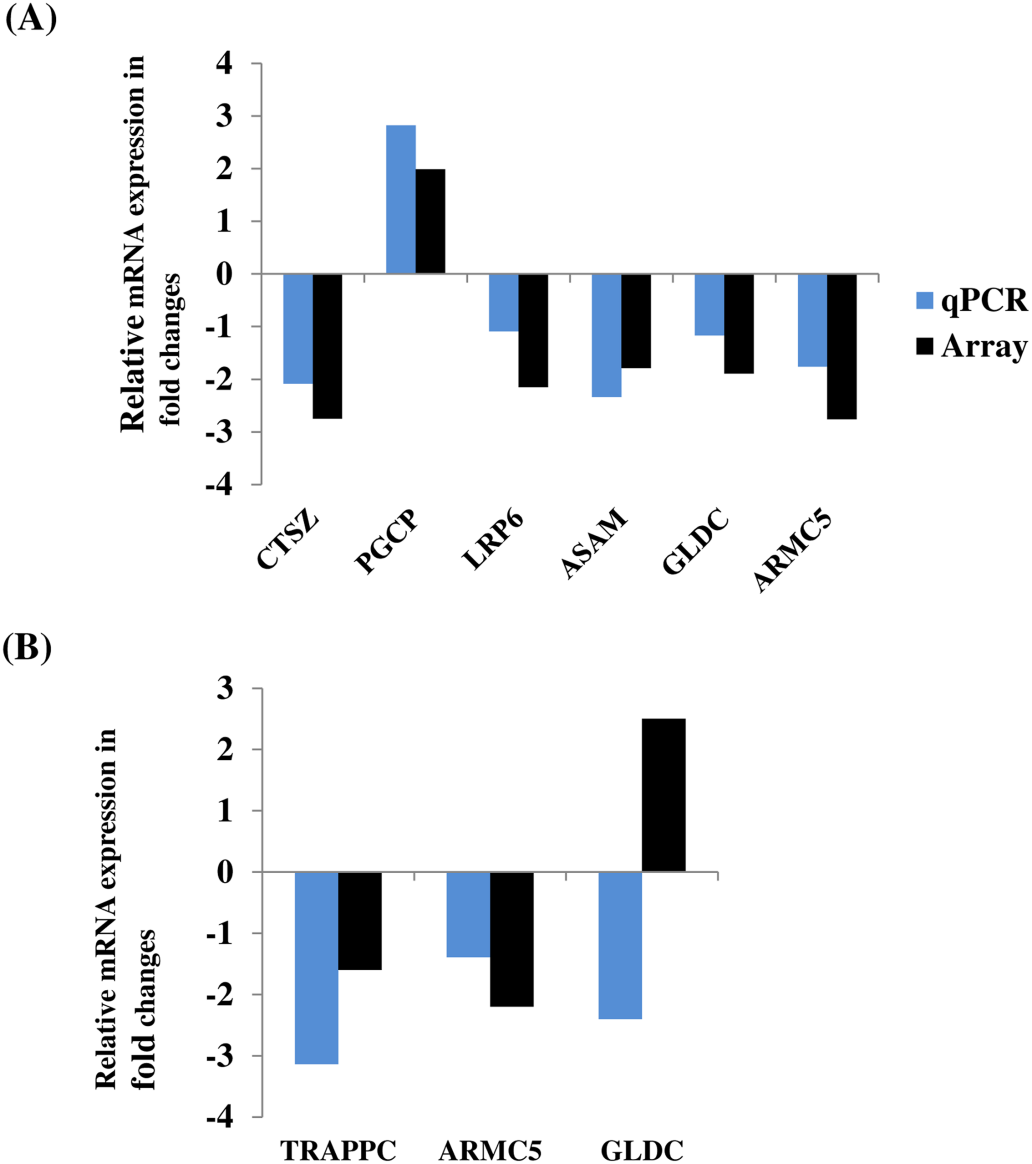
The qPCR validation result of selected candidate differentially expressed genes in IVP (A) and 4C (B) blastocyst groups.

Since comparative analysis of DNA methylation pattern and gene expression of the blastocyst groups was performed by superimposing the current DNA methylation data and our previouly published transcriptome profile data [22], we sought to confirm the expression pattern of selected candidate differentially expressed genes in RNA samples isolated from blastocysts which were used for DNA methyltion analysis. The result indicated that the expression profile of 6 of the 7 selected genes was found to be in line to the array data (Fig 13).

## Discussion

Most often, in vitro produced embryos exhibited a reduced quality and viability compared to their in vivo counter parts. The molecular data also indicated compromised gene transcription, imprinting gene maintenance errors and repression of transposable elements in in vitro produced embryos due to altered epigenetic regulation such as aberrant DNA methylation [31,32]. However, it has been a challenge to identify the embryonic stages that could activate aberrant DNA methylation patterns when embryos are cultured under suboptimal culture conditions. Here, we investigated the genome wide DNA methylation patterns of blastocysts developed in vivo after preimplantation embryos were developed in vitro until zygote (ZY), 4-cell (4C) or 16-cell (16C) stages. The blastocysts developed completely under in vitro culture condition (IVP) were also included in the study to understand the effect of continuous in vitro culture on the DNA methylation pattern of the blastocyst stage bovine embryo. The DNA methylation pattern of all blastocyst groups were determined using blastocysts developed completely under in vivo condition (VO) as a common reference. Interestingly, we have identified a higher DNA methylation dysregulation in the IVP blastocysts followed by the 16C and 4C blastocyst groups, and relatively lower DNA methylation dysregulation was detected in the ZY blastocyst group. Therefore, the extent of DNA methylation dysregulations of the blastocysts seem to be strongly associated with the successive developmental stages completed under in vitro condition (Fig 1). In line to this, our previous transcriptome profile data has also indicated a higher gene expression dysregulation in IVP and 16C blastocyst groups than the 4C blastocyst group [22]. However, the critical question is: why differentially methylated genomic loci were increased in the resulting blastocysts when the preimplantation embryos advanced their development stages under in vitro culture conditions? One hypothesis is that, any stage development the embryo undergoes may be associated with the induction of a fixed level of methylation dysregulation, and early transferred embryos to the in vivo condition could have relatively more time to correct that dysregulation and revert to the normal state. Therefore, culturing embryos until blastocyst stage in vitro could induct dysregulation of DNA methylation profile in many more genomic loci than those blastocysts derived from embryos cultured in vitro only until certain specific stages of development.

Despite differences in the stage of development in which the embryos spent under in vitro environment, certain genomic regions were commonly differentially methylated in two or more blastocyst groups (Fig 3). For instance, although, the ZY; 4C and 16C blastocyst groups were derived from preimplantation stages embryos exposed to in vitro culture until certain stages, 26% (66/253), 29.8% (290/ 974) and 3% (45/1245) of the DMRs in ZY, 4C and 16C blastocyst groups were found to be detected in the IVP blastocyst group indicating relatively higher communality between the 4C and IVP blastocyst groups. In fact, the 16C blastocysts were derived from embryos developed in vivo after transfer of in vitro produced 16-cell embryos, which the EGA occurred in vitro, intuitively, higher commonality would be expected between the 16C and IVP than the 4C and IVP blastocyst groups. This may in turn suggest that reprogramming errors occurred due to suboptimal culture condition before the major embryonic genome activation may prevail until the blastocyst stage although major embryonic genome activation occurs under in vivo condition. Our previous transcriptome profile data also indicated that higher communality in number of differentially expressed genes between blastocyst of the IVP and the 4C groups than IVP and 16C [22]. Then the core question is why the 4-cell transfer group tended to share relatively higher number of DMRs with IVP than the 16C blastocyst group did? Although further research is required to deduce a conclusion, this can be associated with the epigenetic memory that could be remained inscribed due to suboptimal environmental condition before embryonic genome activation. In bovine embryos, although minor embryonic genome activation (EGA) may start at 2-to 4-cell stage [33–35], major EGA is believed to occur at the 8- to 16-cell stages [14,36–39]. For instance, Kues et al. [39] indicated increased expression level of about 2400 genes in 8-cell embryos compared to 2-cell and/or 4-cell stages and Jiang et al. [38] reported also significantly higher number of differentially expressed genes as the embryo grows 4 to 8-cell stages suggesting in both cases the occurrence of major EGA at the 8-cell stage. In fact, activation of the maternal genome and transcription of the genome messages could be partly control by the pattern of DNA methylation [40]. In this regards in vitro studies indicated that the bovine embryos undergo global DNA methylation erasure soon after fertilization until 8 cell stage and an increase in methylation level occurs at 16-cell until blastocyst stage [13,15,41]. Therefore, we can speculate that some reprogramming errors that could occur before major embryonic genome activation could be displayed at the blastocyst stage although the embryos were set into the optimal culture condition.

In addition to looking into the general trends of the differentially methylated genomic regions, we have also sought to explore the genomic features whose methylation patterns were affected by the in vitro culture condition. To this end, we have also analyzed the differentially methylated CpG islands, which are believed to be the evolutionary landmarks of molecular events occurring at eukaryotic promoters [42]. Indeed, along with other genomic features, the CpG islands were also differentially methylated in all blastocyst groups although the ZY, 4C and 16C blastocyst groups were relatively less affected compared to the IVP group. Since differences in the length of the CpG islands can be associated the functional changes, we have also explored the characteristics of the differentially methylated region with respect to its CpG island length, [43]. In this regard, our result indicated differences in the proportion of long, intermediate and short CpG islands between the blastocyst groups of which the frequency of small length differentially methylated CpG islands were dominant in the 4C blastocyst group, while intermediate length CpG islands were dominant in ZY, 16C and IVP blastocyst groups (Fig 4B). This may suggest that the DNA methylation pattern of genomic regions dominated by short or long stretches of CpG sites in the blastocysts could be determined by the stage of development the embryos completed under in vitro culture condition.

Exploring the genomic region of differentially methylated CpG islands and examining the effect of these genomic features on gene expression of nearby genes may spark light of elimination to get an insight into the effect of suboptimal embryo culture condition on the embryo methylene. Indeed, our data evidenced that unlike to the ZY and IVP groups, the majority of differentially methylated CpG islands in 4C and 16C blastocyst groups were hypermethylated. However, only few hypermethylated CpG islands were located on the promoter region of these blastocyst groups. Although most CpG islands are believed to be methylation free [44], parts of the CpG islands could be methylated during development and gene silencing may occur if this happens at the promoter region [41,45–49]. Nevertheless, in the current study, there was a hint that the gene regulation by CpG islands is not limited only at the promoter regions, but also this can happen at the intronic and exonic regions (S7 Table). On other hand, the hypo or hypermethylated CpG islands may not always associated with repression or induction of gene expression (S8 Table).

We have also assessed the location of the non-CpG-island DMRs with respect to the nearest CpG islands to get an overview on the methylation pattern of the genomic regions which are adjacent or far from the CpG islands (Fig 4C and 4D). This type of analysis has been suggested to be useful to correlate the DNA methylation pattern with the corresponding phenotype changes [50]. In the present study, enrichment of differentially methylated regions in CpG shore, Open Sea or CpG shelf tended to be different between the ZY, 4C, 16C and IVP blastocyst groups. For instance, more than 2 fold enrichment of hypermethylated than expected was detected at the CpG shore in 4C and CpG shelf in the 16C blastocyst groups. Indeed, it is suggested that altered DNA methylation profile particularly at CpG island shores is not only regulate the gene expression patterns [51], but also it affects the cell differentiation and development [52]. Generally, characterizing differentially methylated regions with respect to the CpG islands could provide insights into the presence/absence and the level of aberrant DNA methylation at the CpG island and its vicinity. Moreover, describing differentially methylated regions in relation to proximal promoter, promoter, intron and exon is essential to better understand the embryo genome structures subjected to aberrant methylation patterns due to suboptimal culture condition. In this regard, the current study has evidenced the presence of strong difference between the ZY, 4C, 16C and IVP blastocyst groups with respect to genomic enrichment of hypermethylated genomic region. For instance, as high as four fold enrichment of hypermethylated genomic region were detected at proximal promoter and exonic region in 4C group and promoter and proximal promoters in the 16C blastocyst group. However, this was not the case in ZY blastocyst group, of which hypermethylation was favored and in the IVP blastocyst group, of which the ratios of hypermethylated and hypomethylated region in gene body categories, promoter and proximal promoters were close to 1 (which is Log_2_ enrichment = 0) (Fig 6). Here the fundamental question is why blastocysts developed in vivo from in vitro produced 4-cell and 16-cell were associated with hypermethylation whereas the majority of genomic features in blastocysts developed in vivo from in vitro produced zygotes were enriched by hypomethylation and as much hypermethylation as hypomethylation were detected in blastocysts developed completely under in vitro condition in several genomic features? Although, detail and locus specific DNA methylation study may be required to uncover this puzzle, previous studies based on immunoreactive 5-methylcytosine antibody on in vitro culture bovine embryos indicated demethylation of the paternal genome in zygotes and a declining methylation pattern from cleavage until 8-cell stage and increasing methylation level in 16-cell until blastocyst stage [12,13,15].

Parallel to DNA methylation profiling, we have also compared the gene expression data [22] and the current methylation profile to get insight into the association of methylation profile and gene expression patterns in these blastocyst groups. The data revealed that 1.6%, 3.4% 3.9% and 9.4% of the total DMRs mapped to the transcriptome data in ZY, 4C, 16C and IVP, respectively displayed a negative correlation with the corresponding gene expression patterns and the rest exhibited unexpected patterns. Thus, the relationship between the DNA methylation pattern and mRNA expression level was not always as expected provided the negative correlation between gene expression and DNA methylation is the expected trend. In fact, from the DNA methylation pattern of bovine placenta, previously it has been suggested that the relationship between gene-body DNA methylation and gene expression is non-monotonic [53]. Indeed, in several occasions it has been noted that the DNA methylation is negatively correlated with gene expression at the promoter region [45,54,55] and positively correlation at the gene body region [55,56]. However, in our result, it is indicated that some of the gene body methylation were negatively and some others were positively correlated with the gene expression and the same was true for promoter regions (Figs 9, 10 and 11, S5 & S6 Figs). Thus, it is less likely to generalize that methylation in promoter regions typically leads to decreased gene expression while DNA methylation in gene bodies is often correlates with increased gene expression. In line to our study, the study by Yang et al. [57] has also indicated a negatively correlation between some gene body DNA methylations and gene expression. These authors also added that the methylation pattern of only 25 out of 105 probes at the promoter region displayed a significant inverse correlation with the gene expression stressing that even methylation in promoter region may not always result in a decrease in gene expression. Thus, the relationship between gene body/promoter methylation and gene expression may vary depending on the physiological status and the cell type under consideration. On the other hand, looking at single base resolution level may be essential to understand the association between methylation level and gene expression more precisely. For instance, it was reported that non-CG methylation level within the gene body to be positive correlated with the gene expression and however, there was no correlation between CG methylation density and gene expression [58]. However, the main issue should rather focus on extent/ proportion of DMRs that positively or negatively correlated with gene expression. Indeed in our study, it was indicated that only 4.3%, 3.5%, 4.1% and 5.6% of the total DMRs in ZY, 4C, 16C and IVP blastocyst groups were positively or negatively correlated with the corresponding gene expression and the majority of the DMRs didn’t show changes in the gene expression patterns. Nevertheless, it is also known that the DNA methylation is not only regulates the mRNA expression, but also the expression of other non coding RNAs including miRNAs [59–61]. Thus, the DMRs which were not associated with the altered gene expression in ZY, 4C, 16C and IVP blastocyst groups could be involved in regulating small non coding RNAs molecules. Similarly, we have also identified altered gene expressions in ZY, 4C, 16C and IVP blastocyst groups which were not directly associated with DNA methylation dysregulation. In this case, other key epigenetics effectors such as histone modification, chromatin remodeling and non-coding RNAs could be involved to fine tune the regulation of gene expression during embryo development under suboptimal environmental condition.

## Conclusion

In this study, we have provided detailed insights into the DNA methylation landscape of bovine blastocysts developed from embryos cultured under in vitro condition in a stage specific manner before transfer to the in vivo condition along with blastocysts developed completely developed under in vitro condition. The data has shown remarkable differences in the extent of DNA methylation profile of blastocysts depending on developmental stages completed under in vitro culture condition. The DNA methylation patterns of CpG islands and repetitive elements were also affected in the blastocysts depending on the stage of the embryo subjected to the in vitro culture. Blastocysts developed in vivo after transfer of in vitro developed 4- and 16-cell stage embryos displayed increased proportion of hypermethylated genomic loci in the gene body, promoter or proximal promoter regions. Moreover, 1.6, 3.4, 3.9 and 9.4% of the DMRs which overlapped to the transcriptome profile data in ZY, 4C, 16C and IVP blastocyst groups, respectively were negatively correlated with their corresponding gene expression while the majority exhibited unexpected patterns given that DNA methylation is usually thought to negatively regulate gene expression. Therefore, the observed systematic DNA methylation profile alteration in the blastocysts is a clear demonstration the effect of stage specific in vitro culture condition on the embryo epigenome.

## Supporting Information

S1 Fig The number of detected probes which were specific to each sample or common for two or more sample groups. (TIF)

S2 Fig The circos plot representing overall methylation levels in ZY blastocyst group. The mean p-values of 5 Mbp windows are indicated along with the 100 most significant DMRs. Positive and negative fold-changes represent hypermethylation and hypomethylation in ZY blastocyst group relative to VO blastocyst group.
(TIF)

S3 Fig The circos plot representing overall methylation levels in 4C blastocyst group. The mean p-values of 5 Mbp windows are indicated along with the 100 most significant DMRs. Positive and negative fold-changes represent hypermethylation and hypomethylation in 4C blastocyst relative to VO blastocyst group.
(TIF)

S4 Fig The circos plot representing overall methylation levels in 16C blastocyst group. The mean p-values of 5 Mbp windows are indicated along with the 100 most significant DMRs. Positive and negative fold-changes represent hypermethylation and hypomethylation in 16C blastocyst group relative to VO blastocyst group.
(TIF)

S5 Fig Positive correlation between differentially expressed genes and their corresponding DNA methylation profile in ZY (A), 4C (B) and 16C (C) blastocyst groups. DMRs = differentially methylated regions, DEGs = differentially expressed genes. Region = genomic location of the DMRs. Ppromoter = proximal promoter.
(TIF)

S6 Fig Positive correlation between differentially expressed genes and their corresponding DNA methylation profile in IVP blastocyst group. DMRs = differentially methylated regions, DEGs = differentially expressed genes. Region = genomic location of the DMRs. Ppromoter = proximal promoter.
(TIF)

S7 Fig The bisulfite sequencing result indicating the CpG wise DNA methylation pattern of 8 candidate genomic regions in different blastocyst groups. (TIF)

S1 Table The number of embryos transfers to the recipients and blastocyst recovery rates. (DOCX)

S2 Table List of genes and corresponding primers used for validatation of differenitally expressed genes. (DOCX)

S3 Table Commonly differentially methylated regions in ZY, 4C and IVP blastocyst groups. Log_2_FC = level of hypermethylation or hypomethylation in log_2_ scale relative to the VO blastocyst group.
(DOCX)

S4 Table Commonly differentially methylated regions in ZY and 4C blastocyst groups. Log_2_FC = level of hypermethylation or hypomethylation in log_2_ scale relative to the VO blastocyst group.
(DOCX)

S5 Table Differentially methylated regions in 4C and IVP blastocyst groups. Log_2_FC = hyper or hypomethylation in log_2_ scale relative to VO blastocyst group, Ppromoter = proximal promoter, Dpromoter = Distal promoter.
(XLSX)

S6 Table The list of all differentially methylated CpG islands in IVP blastocyst group. Log_2_FC = hypermethylation or hypomethylation in log_2_ scale relative to the VO blastocyst group, Ppromoter = proximal promoter, Dpromoter = Distal promoter.
(XLSX)

S7 Table List of differentially methylated CpG islands exhibited inverse correlation with their corresponding gene expression in IVP blastocyst group. Interm. = intermediate islands, Ppromoter = Proximal promoter.
(DOCX)

S8 Table Positive correlation between the differentially methylated CpG islands and their corresponding gene expression in IVP blastocyst group. Interm. = intermediate islands.
(DOCX)
