# Supplementary figures and images for: Genome-Wide DNA Methylation Patterns of Bovine Blastocysts Developed In Vivo from Embryos Completed Different Stages of Development In Vitro
Source: PLoS One. 2015 Nov 4;10(11):e0140467. doi: 10.1371/journal.pone.0140467 (PMC4633222; doi:10.1371/journal.pone.0140467)

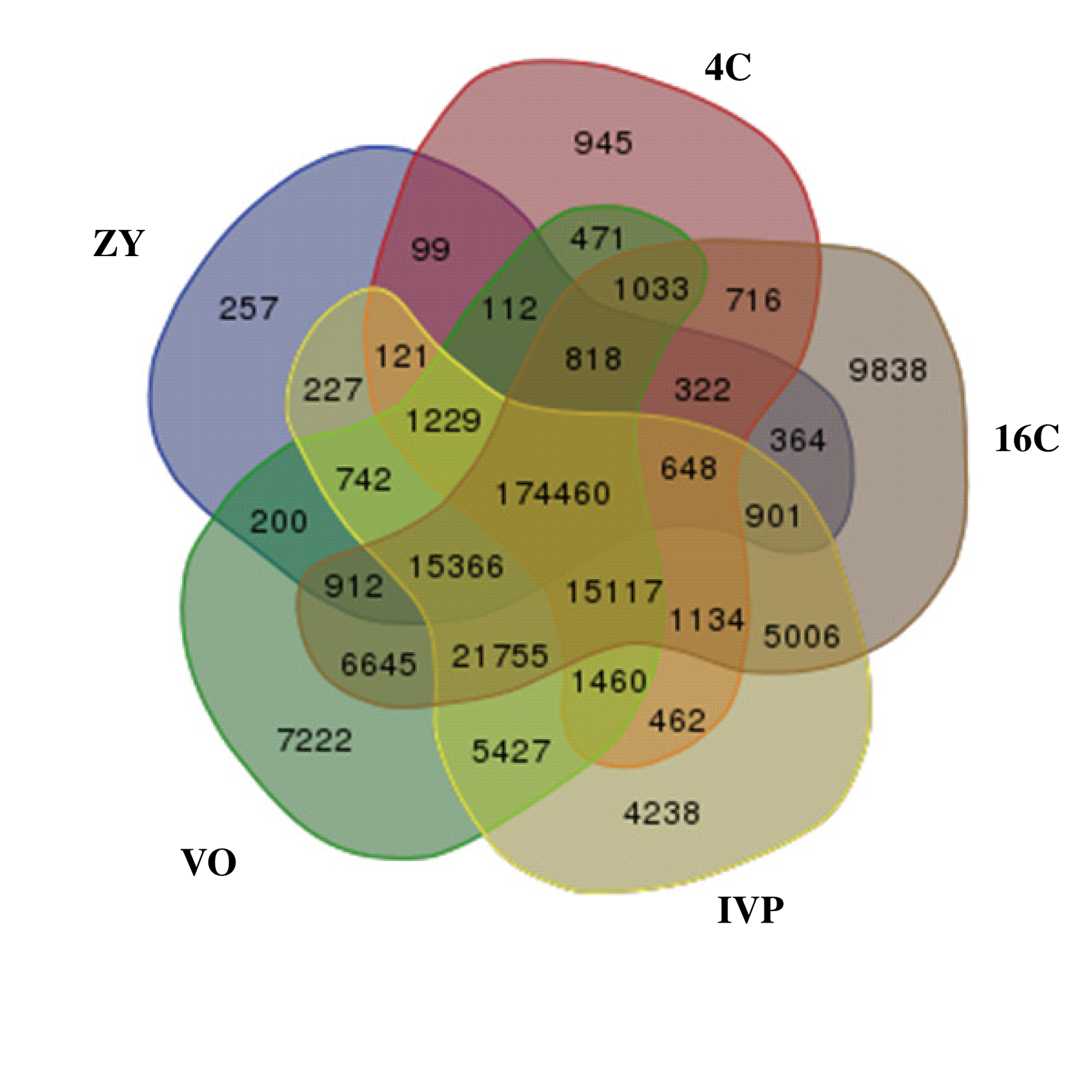

Supplement: S1 Fig — (TIF) [file pone.0140467.s001.tif]

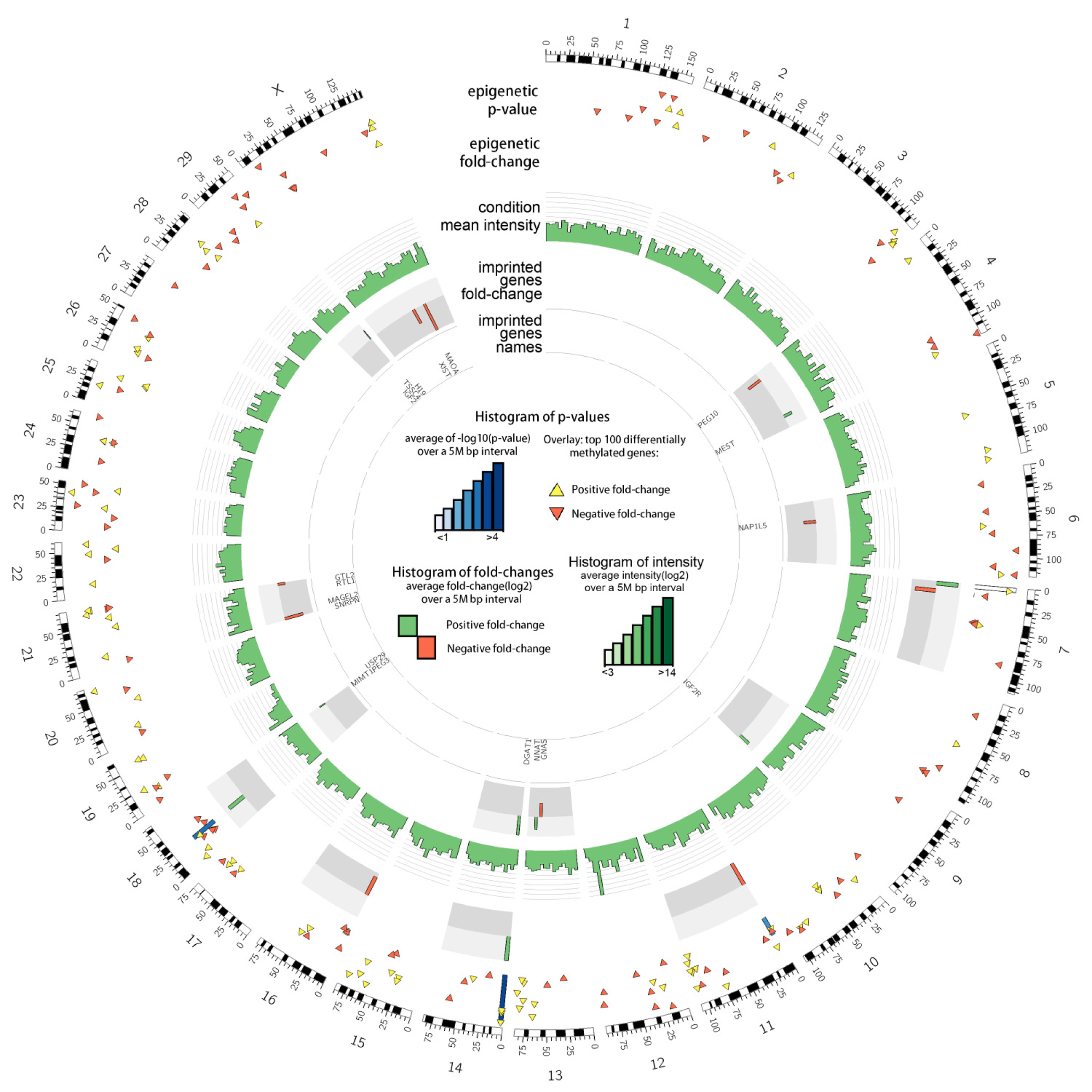

Supplement: S2 Fig — The mean p-values of 5 Mbp windows are indicated along with the 100 most significant DMRs. Positive and negative fold-changes represent hypermethylation and hypomethylation in ZY blastocyst group relative to VO blastocyst group. (TIF) [file pone.0140467.s002.tif]

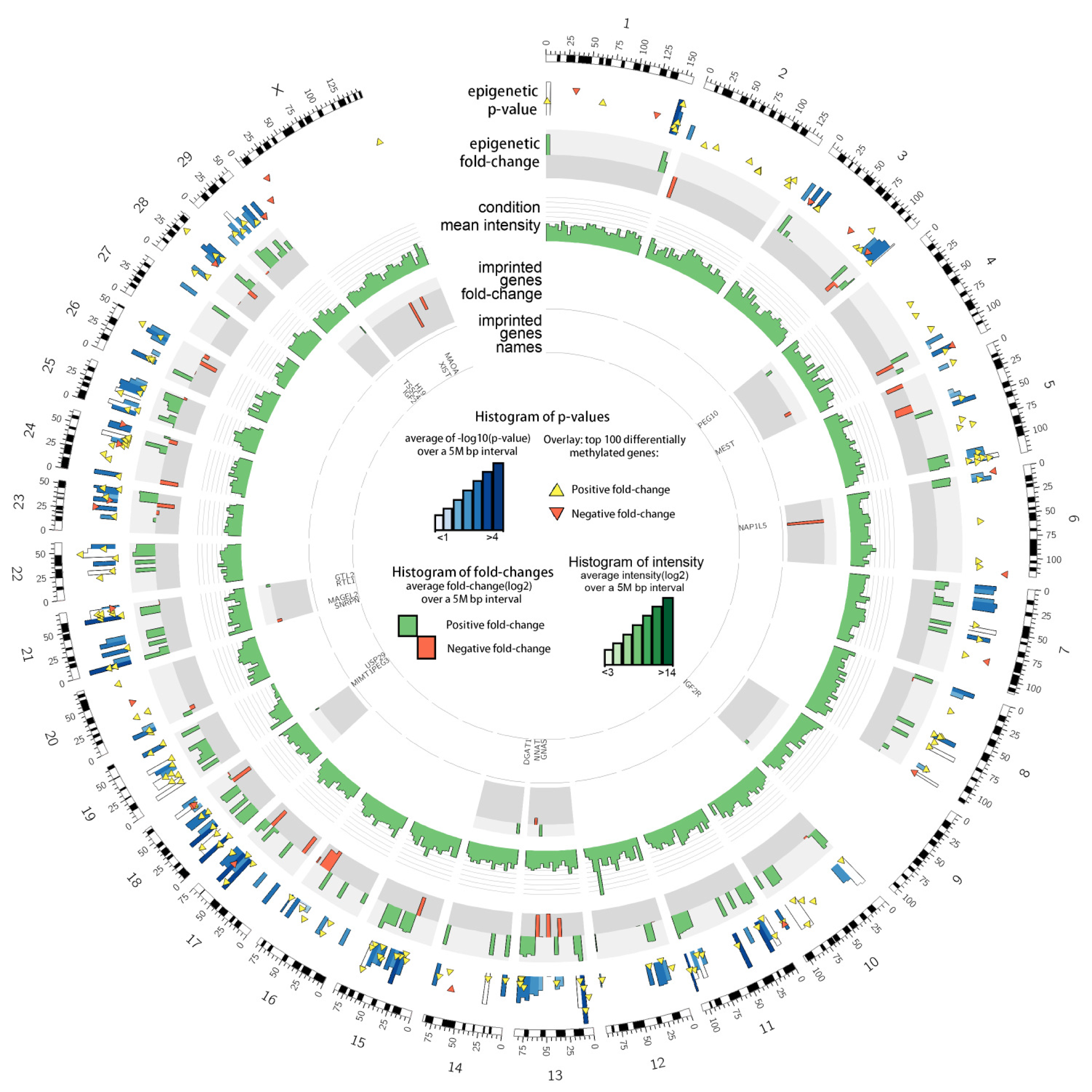

Supplement: S3 Fig — The mean p-values of 5 Mbp windows are indicated along with the 100 most significant DMRs. Positive and negative fold-changes represent hypermethylation and hypomethylation in 4C blastocyst relative to VO blastocyst group. (TIF) [file pone.0140467.s003.tif]

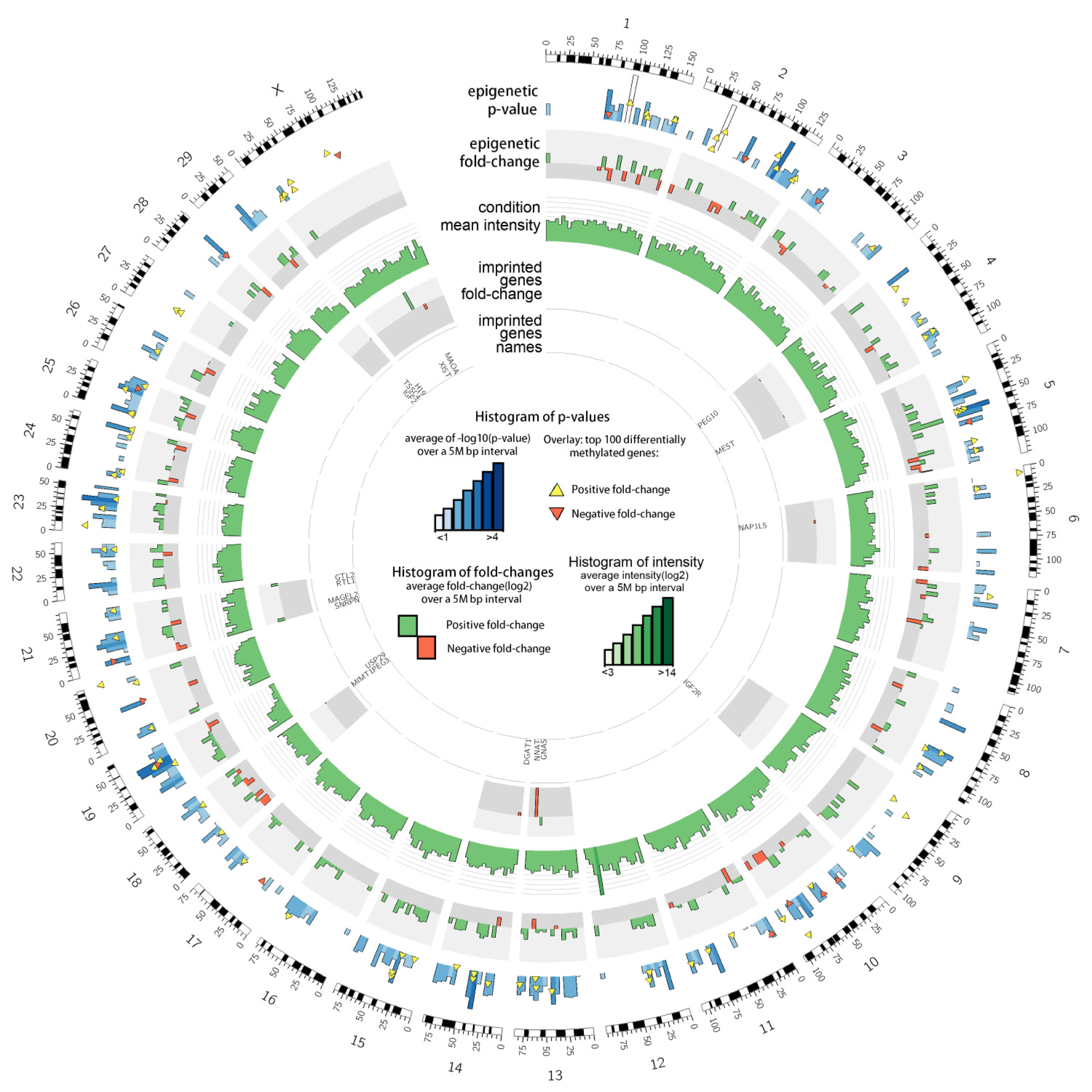

Supplement: S4 Fig — The mean p-values of 5 Mbp windows are indicated along with the 100 most significant DMRs. Positive and negative fold-changes represent hypermethylation and hypomethylation in 16C blastocyst group relative to VO blastocyst group. (TIF) [file pone.0140467.s004.tif]

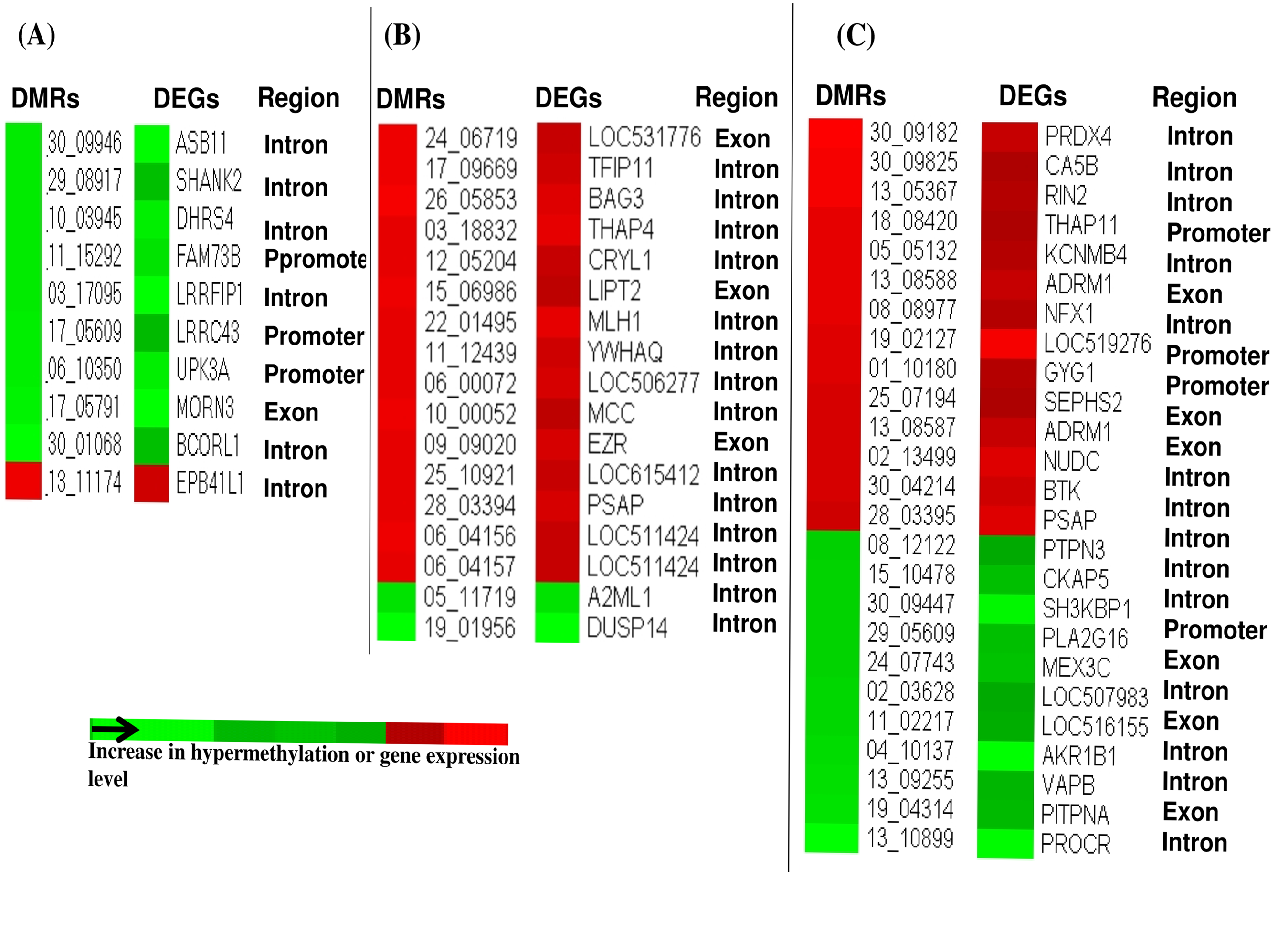

Supplement: S5 Fig — DMRs = differentially methylated regions, DEGs = differentially expressed genes. Region = genomic location of the DMRs. Ppromoter = proximal promoter. (TIF) [file pone.0140467.s005.tif]

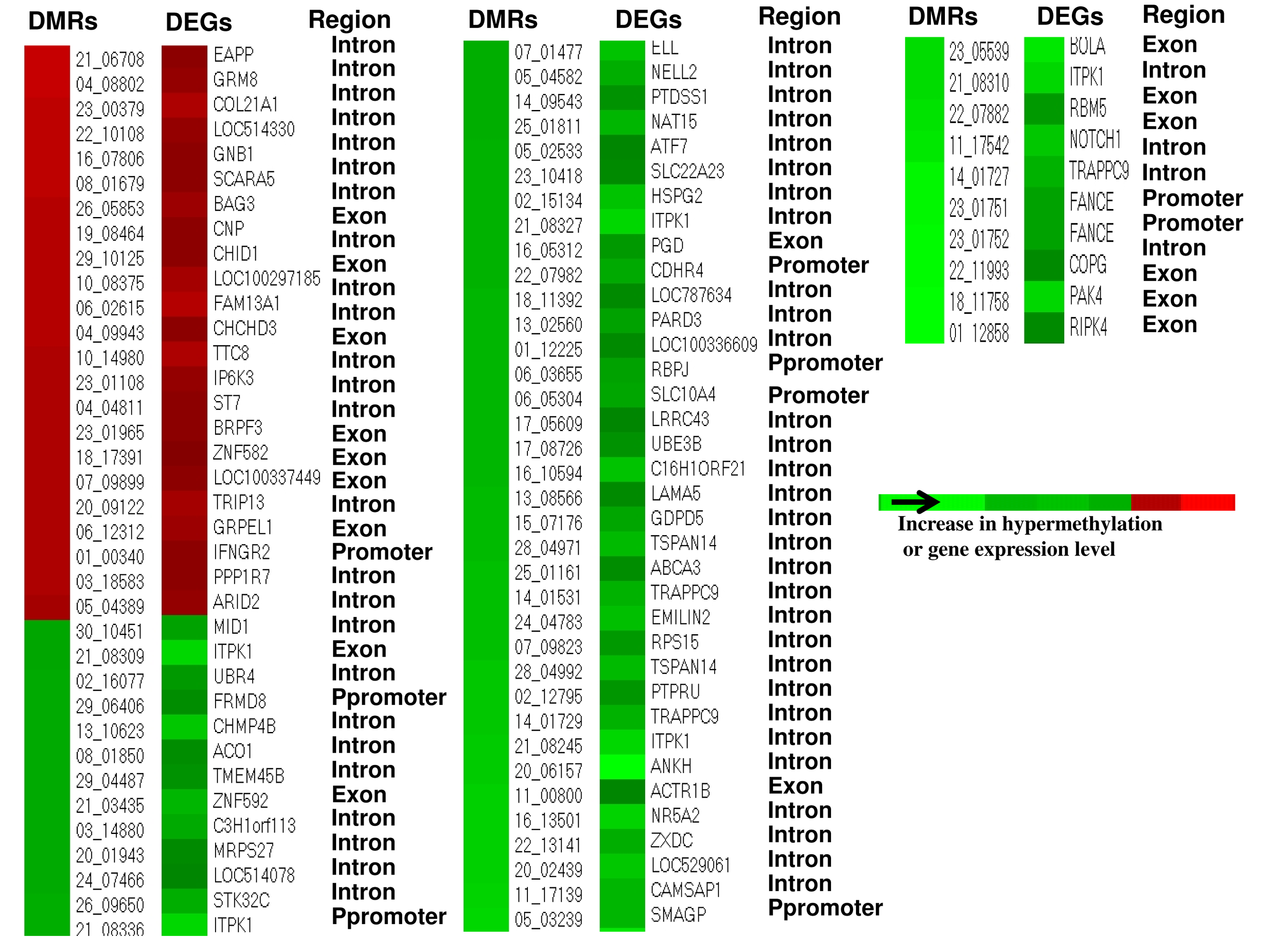

Supplement: S6 Fig — DMRs = differentially methylated regions, DEGs = differentially expressed genes. Region = genomic location of the DMRs. Ppromoter = proximal promoter. (TIF) [file pone.0140467.s006.tif]

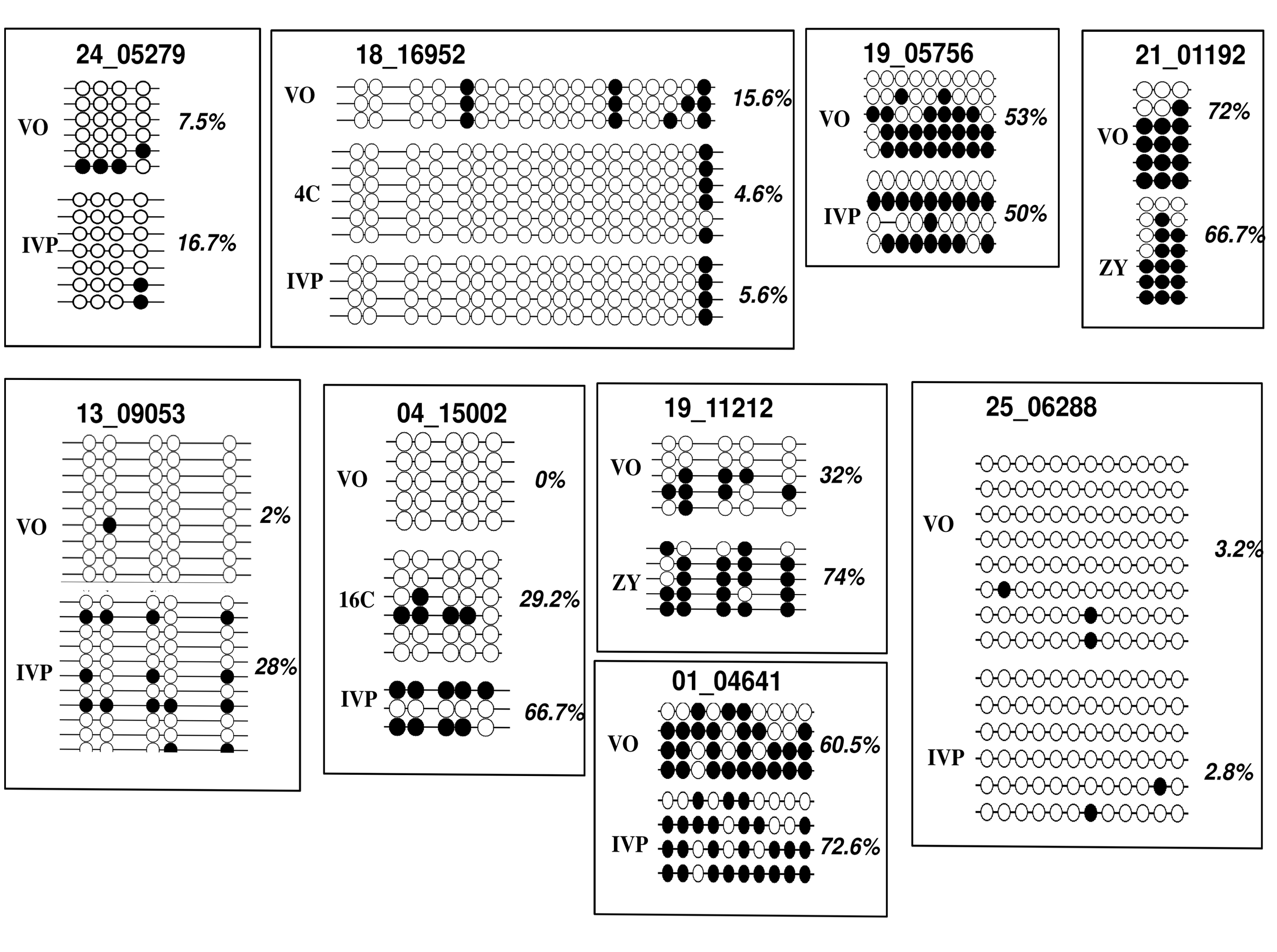

Supplement: S7 Fig — (TIF) [file pone.0140467.s007.tif]
