# Supplementary material for: Genome-Wide DNA Methylation Patterns of Bovine Blastocysts Developed In Vivo from Embryos Completed Different Stages of Development In Vitro
Source: PLoS One. 2015 Nov 4;10(11):e0140467. doi: 10.1371/journal.pone.0140467 (PMC4633222; doi:10.1371/journal.pone.0140467)
Supplement: S1 Table — (DOCX) [file pone.0140467.s008.docx]

| **Group** | **Recipients**  **(n)** | **Embryos**  **transferred / recipient** | **Total transferred embryos** | **Recollected embryos** | | **Blastocysts** | | |
| --- | --- | --- | --- | --- | --- | --- | --- | --- |
|  |  |  |  |  |  | **total** | **per recollected** | **per total** |
|  |  | **(n)** | **(n)** | **(n)** | **(%)** | **(n)** | **(%)** | **(%)** |
| Zygote-transfer | 6 | ~ 100 | 600 | 404 | 67.3% | 76 | 18.8% | 12.7% |
| 4-cell transfer | 5 | ~ 20 | 105 | 90 | 85.7% | 43 | 47.8% | 41.0% |
| 16-cell transfer | 4 | ~ 35 | 138 | 121 | 87.7% | 77 | 63.6% | 55.8% |

**S1.Table: The number of embryos transfer to the recipients and blastocyst recovery rates.**
