# Supplementary material for: Genome-Wide DNA Methylation Patterns of Bovine Blastocysts Developed In Vivo from Embryos Completed Different Stages of Development In Vitro
Source: PLoS One. 2015 Nov 4;10(11):e0140467. doi: 10.1371/journal.pone.0140467 (PMC4633222; doi:10.1371/journal.pone.0140467)
Supplement: S2 Table — (DOCX) [file pone.0140467.s009.docx]

| Gene ID | Gene symbol | Gene title | 5’-3’ | Bp |
| --- | --- | --- | --- | --- |
| NM_001077835.1 | *CTSZ* | Bos taurus cathepsin Z | F GATCCCATTGTCACAGGCAC  R GAGGTCACATCGTTCACTGC | 220 |
| XM_003586111.2 | *LRP6* | PREDICTED: *Bos taurus* low density lipoprotein receptor-related protein 6 transcript variant X2 | F TACTGGACAGACTGGGGAGA  R GGCAAAAGGATGTGGAAGGG | 230 |
| XM_002698104.2 | *ARMC5* | PREDICTED: *Bos taurus* armadillo repeat containing 5 | F CAACTGCTGTACTGAAGGGG  R GCTCTCAGGTTCCATGGCTA | 150 |
| NM_001076248.1 | *PGCP*  *( CPQ)* | *Bos taurus* plasma glutamate carboxypeptidase | F GACGCAGAGATGATGTCAAGA  R GATATAAAGGCTCCACCACCA | 217 |
| XM_005215951.1 | *CLMP (ASAM)* | PREDICTED: *Bos taurus* CXADR-like membrane protein , transcript variant X1 | F TCTGAACCAAAGAAAGCCCC  R TGCTGCTACTTAAATGACCCC | 216 |
| NM_001192951.1 | *GLDC* | Bos taurus glycine dehydrogenase (decarboxylating) (GLDC), mRNA | F TGATCGAGAAGACCATCCCTG  R AGTTCTCCAGTAAGTTCCGCA | 178 |
| XM_005215216.2 | *TRAPPC9* | Bos taurus trafficking protein particle complex 9 (TRAPPC9), transcript variant X4, mRNA | F AGACATTACAAGAAGCGGTGC  R AGTGATAGATGACAGAGGCCG | 151 |
| NM_001034034.2 | *GAPDH* | Bos taurus glyceraldehyde-3-phosphate dehydrogenase (GAPDH), mRNA | F CCAGGGCTGCTTTTAATTCT  R ATGGCCTTTCCATTGATGAC | 166 |

**S2 Table. List of genes and corresponding primers used for validatation of differenitally expressed genes.**
