# Supplementary material for: Genome-Wide DNA Methylation Patterns of Bovine Blastocysts Developed In Vivo from Embryos Completed Different Stages of Development In Vitro
Source: PLoS One. 2015 Nov 4;10(11):e0140467. doi: 10.1371/journal.pone.0140467 (PMC4633222; doi:10.1371/journal.pone.0140467)
Supplement: S3 Table — Log2FC = level of hypermethylation or hypomethylation in log2 scale relative to the VO blastocyst group. (DOCX) [file pone.0140467.s010.docx]

| **S3 Table. Commonly differentially methylated regions in ZY, 4C and IVP blastocyst groups**. | **ZY** | | **4C** | | **IVP** | | **Gene symbol** | **Gene region affected** |
| --- | --- | --- | --- | --- | --- | --- | --- | --- |
| **Probe ID** | **Log_2_ FC** | **P-value** | **Log_2_ FC** | **P-value** | **Log_2_**  **FC** | **P-value** |  |  |
| 13_15059 | 1.14 | 0.0082 | 0.75 | 0.0001 | 0.66 | 0.0040 |  |  |
| 28_04337 | 0.81 | 0.0003 | 1.12 | 0.0000 | 0.59 | 0.0021 | *KCNMA1* | Intron |
| 14_00677 | 0.78 | 0.0000 | 0.86 | 0.0012 | 0.76 | 0.0001 |  |  |
| 15_10952 | 0.78 | 0.0001 | 0.63 | 0.0003 | 0.78 | 0.0000 |  |  |
| 14_00675 | 0.76 | 0.0001 | 0.87 | 0.0006 | 0.68 | 0.0001 |  |  |
| 25_05674 | 0.76 | 0.0000 | 0.95 | 0.0000 | 0.78 | 0.0000 | *OTOA* | Intron |
| 24_04879 | 0.74 | 0.0019 | 0.97 | 0.0001 | 0.77 | 0.0001 |  |  |
| 14_00676 | 0.73 | 0.0001 | 0.88 | 0.0012 | 0.64 | 0.0003 |  |  |
| 17_08824 | 0.70 | 0.0023 | 0.68 | 0.0013 | 0.91 | 0.0001 | *ACACB* | Intron |
| 22_07151 | 0.69 | 0.0004 | 0.88 | 0.0000 | 0.94 | 0.0001 | *LOC514296* | Intron |
| 11_19271 | 0.69 | 0.0012 | 0.82 | 0.0000 | 0.60 | 0.0007 |  |  |
| 22_09575 | 0.65 | 0.0149 | 0.97 | 0.0001 | 0.92 | 0.0000 | *ATP2B4* | Intron |
| 10_07331 | 0.64 | 0.0087 | 0.59 | 0.0153 | 0.59 | 0.0003 |  |  |
| 21_07056 | 0.64 | 0.0053 | 0.80 | 0.0002 | 0.79 | 0.0002 | *SLC25A21* | Intron |
| 26_00898 | 0.64 | 0.0008 | 0.86 | 0.0013 | 0.90 | 0.0001 | *CPEB3* | Intron |
| 28_04529 | 0.62 | 0.0057 | 0.68 | 0.0007 | 0.81 | 0.0000 |  |  |
| 13_14065 | 0.61 | 0.0057 | 0.81 | 0.0041 | 0.80 | 0.0005 |  |  |
| 01_15893 | 0.60 | 0.0040 | 0.71 | 0.0001 | 0.62 | 0.0027 | *RFTN1* | Intron |
| 12_03898 | 0.60 | 0.0012 | 0.79 | 0.0001 | 0.91 | 0.0001 | *LOC616677* | Intron |
| 11_00282 | 0.60 | 0.0022 | 0.75 | 0.0000 | 1.08 | 0.0001 | *LOC100336467* | Intron |
| 23_05207 | 0.59 | 0.0027 | 0.59 | 0.0018 | 0.76 | 0.0001 | *LY6G6C* | Intron |
| 18_07911 | -0.59 | 0.0057 | -0.59 | 0.0123 | -1.32 | 0.0008 |  |  |
| 06_11021 | -0.60 | 0.0017 | -0.74 | 0.0003 | -0.59 | 0.0143 | *WHSC2* | Intron |
| 24_05011 | -0.61 | 0.0027 | -1.01 | 0.0001 | -0.77 | 0.0003 |  |  |
| 07_02466 | -0.64 | 0.0184 | -0.72 | 0.0186 | -1.09 | 0.0014 | *LOC520939* | Intron |
| 07_07534 | -0.64 | 0.0189 | -0.90 | 0.0040 | -0.78 | 0.0179 |  |  |
| 30_00109 | -0.65 | 0.0258 | -1.06 | 0.0001 | -1.75 | 0.0002 |  |  |
| 24_10586 | -0.66 | 0.0002 | -0.68 | 0.0001 | -0.84 | 0.0001 |  |  |
| 26_07309 | -0.68 | 0.0001 | -0.79 | 0.0003 | -0.74 | 0.0004 | *FANK1* | Intron |
| 20_01269 | -0.68 | 0.0028 | -0.63 | 0.0076 | -0.88 | 0.0002 |  |  |
| 20_06157 | -0.69 | 0.0032 | -0.79 | 0.0011 | -0.91 | 0.0179 | *ANKH* | Intron |
| 13_03849 | -0.72 | 0.0136 | -0.70 | 0.0101 | -0.80 | 0.0297 |  |  |
| 19_00275 | -0.75 | 0.0003 | -0.68 | 0.0022 | -0.92 | 0.0000 |  |  |
| 23_05456 | -0.76 | 0.0001 | -0.75 | 0.0003 | -0.89 | 0.0001 |  |  |
| 07_15887 | -0.78 | 0.0001 | -0.73 | 0.0000 | -0.61 | 0.0158 |  |  |

| 28_02919 | -0.79 | 0.0200 | -0.73 | 0.0331 | -0.92 | 0.0321 |  |  |
| --- | --- | --- | --- | --- | --- | --- | --- | --- |
| 12_04847 | -0.79 | 0.0001 | -0.59 | 0.0023 | -0.82 | 0.0003 | SACS9 | Exon |
| 04_10410 | -0.81 | 0.0088 | -0.68 | 0.0281 | -0.69 | 0.0444 |  |  |
| 06_12082 | -0.81 | 0.0001 | -0.78 | 0.0000 | -0.78 | 0.0042 |  |  |
| 01_10974 | -0.82 | 0.0001 | -0.75 | 0.0000 | -0.83 | 0.0017 | *CLSTN2* | Intron |
| 10_16166 | -0.83 | 0.0006 | -0.80 | 0.0000 | -0.78 | 0.0000 | *TTC7B* | Intron |
| 06_06778 | -0.83 | 0.0001 | -0.81 | 0.0017 | -2.74 | 0.0000 | *AREG* | Intron |
| 07_09727 | -0.87 | 0.0120 | -0.82 | 0.0282 | -0.87 | 0.0368 | *MIDN* | Exon |
| 04_13565 | -0.95 | 0.0000 | -0.89 | 0.0000 | -0.99 | 0.0103 | *DPP6-24*  *HTR5A* | Intron |
| 30_01068 | -0.97 | 0.0000 | -0.84 | 0.0000 | -1.20 | 0.0000 | *BCORL1* | Intron |

Log_2_FC= level of hypermethylation or hypomethylation in log_2_ scale relative to VO blastocyst groups.
