# Supplementary material for: Genome-Wide DNA Methylation Patterns of Bovine Blastocysts Developed In Vivo from Embryos Completed Different Stages of Development In Vitro
Source: PLoS One. 2015 Nov 4;10(11):e0140467. doi: 10.1371/journal.pone.0140467 (PMC4633222; doi:10.1371/journal.pone.0140467)
Supplement: S4 Table — Log2FC = level of hypermethylation or hypomethylation in log2 scale relative to the VO blastocyst group. (DOCX) [file pone.0140467.s011.docx]

|  | **ZY** | | **4C** | |  | |
| --- | --- | --- | --- | --- | --- | --- |
| **Probe ID** | **Log_2_ FC** | **P-value** | **Log_2_ FC** | **P-value** | **Gene symbol** | **Gene region affected** |
| 19_11212 | 0.85 | 0.0002 | 0.69 | 0.0000 | FBF1 | Exon |
| 15_07543 | 0.83 | 0.0000 | 0.66 | 0.0004 | MYO7A | Intron |
| 15_08881 | 0.80 | 0.0001 | 1.06 | 0.0001 | LDLRAD3 | Intron |
| 20_07421 | 0.80 | 0.0000 | 0.61 | 0.0020 |  |  |
| 19_04406 | 0.76 | 0.0001 | 0.80 | 0.0000 |  |  |
| 08_06537 | 0.74 | 0.0005 | 0.79 | 0.0001 | **S4Table. Commonly differentially methylated regions in ZY and 4C blastocyst groups.** |  |
| 02_08431 | 0.74 | 0.0003 | 0.90 | 0.0001 |  |  |
| 06_04849 | 0.68 | 0.0060 | 0.81 | 0.0012 |  |  |
| 15_04156 | 0.67 | 0.0006 | 0.75 | 0.0004 | KCNC1 | Intron |
| 25_10875 | 0.66 | 0.0146 | 0.63 | 0.0034 |  |  |
| 15_08354 | 0.65 | 0.0007 | 0.61 | 0.0001 | LOC100300544 | Intron |
| 15_02279 | 0.65 | 0.0003 | 0.62 | 0.0010 |  |  |
| 10_07330 | 0.65 | 0.0042 | 0.70 | 0.0111 |  |  |
| 21_08517 | 0.65 | 0.0026 | 0.71 | 0.0000 | ISG12(A) | Intron |
| 10_11545 | 0.64 | 0.0004 | 0.87 | 0.0000 |  |  |
| 13_14005 | 0.63 | 0.0005 | 0.67 | 0.0001 |  |  |
| 03_17074 | 0.60 | 0.0002 | 0.62 | 0.0001 | LRRFIP1 | Intron |
| 26_01543 | 0.59 | 0.0006 | 0.66 | 0.0043 | PIK3AP1 | Intron |
| 01_15953 | 0.59 | 0.0006 | 0.75 | 0.0000 |  |  |
| 24_05876 | 0.59 | 0.0223 | 0.69 | 0.0077 |  |  |
| 02_01726 | 0.60 | 0.0069 | 0.69 | 0.0064 | LOC100295298 | Intron |
| 21_00730 | 0.61 | 0.0149 | 0.69 | 0.0135 | ADAMTS17 | Intron |
| 03_17035 | 0.61 | 0.0003 | 0.62 | 0.0004 | LRRFIP1 | Intron |
| 18_06283 | 0.61 | 0.0016 | 0.68 | 0.0002 |  |  |
| 08_11289 | 0.61 | 0.0011 | 0.62 | 0.0006 |  |  |
| 03_14474 | 0.61 | 0.0193 | 0.73 | 0.0293 | EPHA10 | Intron |
| 21_11702 | 0.62 | 0.0032 | 0.64 | 0.0001 | LOC100337435 | Promoter |
| 16_00787 | 0.63 | 0.0113 | 0.71 | 0.0181 |  |  |
| 29_08917 | 0.64 | 0.0003 | 0.59 | 0.0001 | SHANK2 | Intron |
| 03_05125 | 0.65 | 0.0276 | 0.63 | 0.0406 | SYT6 | Intron |
| 25_06156 | 0.68 | 0.0007 | 0.63 | 0.0024 |  |  |
| 08_13826 | 0.70 | 0.0170 | 0.64 | 0.0142 | TLR4 | Promoter |
| 16_10144 | 0.70 | 0.0431 | 0.75 | 0.0239 | CACNA1E | Promoter |
| 14_08304 | 0.70 | 0.0002 | 0.83 | 0.0000 | LRP12 | Exon |
| 12_09024 | 0.71 | 0.0168 | 0.76 | 0.0154 |  |  |
| 15_05030 | 0.71 | 0.0378 | 0.75 | 0.0419 |  |  |

| 29_00482 | 0.74 | 0.0099 | 0.71 | 0.0441 |  |  |
| --- | --- | --- | --- | --- | --- | --- |
| 23_03264 | 0.79 | 0.0216 | 0.79 | 0.0162 | CCND3 | Intron |
| 16_04536 | 0.82 | 0.0005 | 0.83 | 0.0042 | FASLG | Exon |
| 01_15990 | 0.82 | 0.0270 | 0.72 | 0.0299 |  |  |
| 13_07247 | 0.96 | 0.0057 | 0.79 | 0.0446 | BMP2 | Intron |
| 14_04273 | 0.99 | 0.0130 | 1.01 | 0.0207 |  |  |
| 11_02815 | 1.07 | 0.0012 | 1.16 | 0.0010 |  |  |
| 23_03593 | 1.23 | 0.0122 | 1.08 | 0.0397 | PTK7 | Intron |

Log_2_FC=level of hypermethylation or hypomethylation in log_2_ scale relative to VO blastocyst group.
