# Supplementary material for: Genome-Wide DNA Methylation Patterns of Bovine Blastocysts Developed In Vivo from Embryos Completed Different Stages of Development In Vitro
Source: PLoS One. 2015 Nov 4;10(11):e0140467. doi: 10.1371/journal.pone.0140467 (PMC4633222; doi:10.1371/journal.pone.0140467)
Supplement: S7 Table — Interm. = intermediate islands, Ppromoter = Proximal promoter. (DOCX) [file pone.0140467.s014.docx]

| Probe Id | CpG island type | Genomic location  of the fragment | Differential methylation | | Genes containing the DMRs | Location of the DMR in the gene | Gene expression | |
| --- | --- | --- | --- | --- | --- | --- | --- | --- |
|  |  |  | Log_2_FC | P value |  |  | Log_2_FC | p value |
| 07_00232 | Interm | 7:1344988-1345335 | -0.86 | 0.0000 | SQSTM1-7 | Intronic | 0.63 | 0.0089 |
| 29_02224 | Interm. | 29:22262760-22263762 | -0.60 | 0.0006 | LOC100300295-1 | Exonic | 0.56 | 0.0051 |
| 29_05401 | Interm. | 29:41683637-41685377 | -0.88 | 0.0012 | INTS5-2 | Exonic | 0.62 | 0.0037 |
| 29_06339 | Interm. | 29:44062208-44063331 | -0.84 | 0.0002 | CAPN1 | Ppromoter | 0.69 | 0.0002 |
| 11_16905 | Interm. | 11:103077174-103077814 | -0.88 | 0.0072 | GTF3C5 | Promoter | 0.62 | 0.0019 |
| 25_14264 | Interm. | 25:42576353-42577284 | -1.38 | 0.0024 | PDGFA-2 | Intronic | 0.91 | 0.0034 |
| 19_04331 | Interm. | 19:23262152-23263555 | 0.70 | 0.0459 | SLC43A2-1 | Exonic | -1.06 | 0.0003 |
| 07_02810 | Interm. | 7:9033661-9034313 | 0.60 | 0.0082 | SYDE1-2 | Exonic | -1.02 | 0.0002 |
| 05_14138 | Small | 5:110449454-110451140 | 0.59 | 0.0025 | PLA2G6-15 | Exonic | -0.88 | 0.0000 |
| 14_06270 | Interm. | 14:36470387-36471172 | -0.60 | 0.0035 | LACTB2-7 | Exonic | 0.65 | 0.0437 |
| 29_06537 | Interm. | 29:44461562-44462670 | -1.52 | 0.0000 | PCNXL3-1 | Exonic | 0.75 | 0.0779 |
| 11_04130 | Interm. | 11:24835989-24836686 | -1.10 | 0.0183 | COX7A2L-3 | Exonic | 0.76 | 0.0018 |
| 06_01157 | Interm. | 6:17710763-17711455 | -0.60 | 0.0045 | AGXT2L1-1 | Exonic | 1.59 | 0.0003 |
| 18_03189 | Interm. | 18:11124182-11124888 | 0.78 | 0.0224 | ZDHHC7 | Promoter | -0.58 | 0.0337 |
| 08_02239 | Interm. | 8:17026951-17027629 | 0.65 | 0.0012 | LOC100335177-9 | Intronic | -1.40 | 0.0012 |
| 24_05279 | Small | 24:40454235-40454472 | 0.83 | 0.0003 | LAMA1-51 | Exonic | -1.44 | 0.0000 |
| 24_05278 | Small | 24:40454235-40454472 | 0.76 | 0.0002 | LAMA1-51 | Exonic | -1.44 | 0.0000 |

**S7 Table. List of differentially methylated CpG islands exhibited inverse correlation with their corresponding gene expression patterns in IVP blastocyst group.**

Interm.= intermediate islands, Ppromoter= Proximal promoter.
