# Supplementary material for: Genome-Wide DNA Methylation Patterns of Bovine Blastocysts Developed In Vivo from Embryos Completed Different Stages of Development In Vitro
Source: PLoS One. 2015 Nov 4;10(11):e0140467. doi: 10.1371/journal.pone.0140467 (PMC4633222; doi:10.1371/journal.pone.0140467)
Supplement: S8 Table — Interm. = intermediate islands. (DOCX) [file pone.0140467.s015.docx]

**S8 Table. Positive correlation between the differentially methylated CpG islands and their corresponding gene expression in IVP blastocyst group.**

| Probe Id | CpG island type | Genomic location  of the fragment | Differential methylation | | Genes containing the DMRs | Location of the DMR in the gene | Gene expression | | |
| --- | --- | --- | --- | --- | --- | --- | --- | --- | --- |
|  |  |  | Log_2_FC | P value |  |  | Log_2_FC | p value | |
| 22_07982 | Interm. | 22:51007858-51011174 | -0.69 | 0.0119 | CDHR4-11-15 | Exonic | -0.85 | 0.0134 | |
| 24_04783 | Interm. | 24:37553996-37555038 | -0.80 | 0.0006 | EMILIN2-6-7 | Exonic | -1.10 | 0.0133 | |
| 18_17391 | Interm. | 18:63941068-63941798 | 0.65 | 0.0073 | ZNF582-1 | Exonic | 0.54 | 0.0119 | |
| 19_08464 | Interm. | 19:42778301-42779167 | 0.69 | 0.0003 | CNP-3 | Exonic | 0.55 | 0.0076 | |
| 22_07882 | Interm. | 22:50813036-50813867 | -1.22 | 0.0004 | RBM5-25 | Exonic | -0.69 | 0.0049 | |
| 20_02439 | Interm. | 20:12560800-12561148 | -0.97 | 0.0000 | LOC529061-27 | Intronic | -1.21 | 0.0049 | |
| 22_13141 | Long | 22:61183331-61183607 | -0.97 | 0.0002 | ZXDC-10 | Intronic | -0.92 | 0.0018 | |
| 10_14980 | Interm. | 10:101570387-101571635 | 0.66 | 0.0107 | TTC8-2 | Exonic | 0.86 | 0.0016 | |
| 22_11993 | Interm. | 22:59807914-59810274 | -1.75 | 0.0000 | COPG-16-18 | Exonic | -0.56 | 0.0011 | |
| 03_14880 | Long | 3:110120249-110120496 | -0.61 | 0.0005 | LOC618094-1-3 | Exonic | -0.86 | 0.0010 | |
| 11_00800 | Interm. | 11:2942738-2943605 | -0.95 | 0.0000 | ACTR1B-13 | Exonic | -0.53 | 0.0008 | |
| 22_10108 | Interm. | 22:56456821-56457368 | 0.79 | 0.0005 | LOC514330-4 | Intronic | 0.66 | 0.0005 | |
| 14_01727 | Interm. | 14:4499244-4500600 | -1.58 | 0.0000 | TRAPPC9-18 | Intronic | -0.97 | | 0.0004 |
| 10_08375 | Interm. | 10:54988599-54988873 | 0.68 | 0.0144 | LOC100297185-2 | Exonic | 0.80 | | 0.0000 |

Interm.= intermediate islands.
